# Amyloid-β impairs mitochondrial dynamics and autophagy in Alzheimer’s disease experimental models

**DOI:** 10.1038/s41598-022-13683-3

**Published:** 2022-06-16

**Authors:** Macarena de la Cueva, Desiree Antequera, Lara Ordoñez-Gutierrez, Francisco Wandosell, Antonio Camins, Eva Carro, Fernando Bartolome

**Affiliations:** 1 Network Center for Biomedical Research in Neurodegenerative Diseases (CIBERNED), Madrid, Spain; 2 Group of Neurodegenerative Diseases, Hospital Universitario 12 de Octubre Research Institute (imas12), 28041 Madrid, Spain; 3 Centro de Biología Molecular “Severo Ochoa” (CSIC-UAM), Universidad Autónoma de Madrid, 28049 Madrid, Spain; 4 Department of Pharmacology, Toxicology and Therapeutic Chemistry, Faculty of Pharmacy & Food Sciences, University of Barcelona, Barcelona, Spain; 5 Institut de Neurociències (UBNeuro), University of Barcelona, Barcelona, Spain

**Keywords:** Biochemistry, Mitochondrial proteins, Animal disease models, Neurological models, Molecular neuroscience, Neuroscience, Cellular neuroscience

## Abstract

The most accepted hypothesis in Alzheimer’s disease (AD) is the amyloid cascade which establishes that Aβ accumulation may induce the disease development. This accumulation may occur years before the clinical symptoms but it has not been elucidated if this accumulation is the cause or the consequence of AD. It is however, clear that Aβ accumulation exerts toxic effects in the cerebral cells. It is important then to investigate all possible associated events that may help to design new therapeutic strategies to defeat or ameliorate the symptoms in AD. Alterations in the mitochondrial physiology have been found in AD but it is not still clear if they could be an early event in the disease progression associated to amyloidosis or other conditions. Using APP/PS1 mice, our results support published evidence and show imbalances in the mitochondrial dynamics in the cerebral cortex and hippocampus of these mice representing very early events in the disease progression. We demonstrate in cellular models that these imbalances are consequence of Aβ accumulation that ultimately induce increased mitophagy, a mechanism which selectively removes damaged mitochondria by autophagy. Along with increased mitophagy, we also found that Aβ independently increases autophagy in APP/PS1 mice. Therefore, mitochondrial dysfunction could be an early feature in AD, associated with amyloid overload.

## Introduction

Alzheimer’s disease (AD) is the most prevalent neurodegenerative disease affecting more than 50 million people worldwide. Patients suffering AD show deep cognitive impairment along with behaviour disorders as the main clinical symptoms. Preceding AD there is a prodromal stage known as mild cognitive impairment (MCI) in which patients still do not show clinical signs of dementia but they undergo the loss of memory, language and other mental abilities with the disease progression. Neuropathologically, AD is characterised by the presence of brain extracellular deposits of amyloid-β (Aβ) peptide coming from the APP processing, the intraneuronal deposits of hyperphosphorylated tau protein, neuroinflammation, and the neuronal cell death in specific brain areas^1,2^. The study of these neuropathological hallmarks gave birth to the main hypothesis to explain the origin of the disease, but nowadays none of them has been totally validated. The most accepted is the amyloid cascade hypothesis which establishes that Aβ oligomeric accumulation is causing AD^3^. This only occurs when APP is processed throughout the amyloidogenic pathway, then producing the Aβ peptide. This accumulation may start years early in the disease progression even years before the main clinical symptoms are evident in patients but the reason because this peptide accumulates is unknown^4^. Along with amyloid accumulation, other possible disease events may occur. Secondary pathological features in AD are evident along with amyloid deposition and they include alterations in the mitochondrial physiology causing energetic deficiency due to mitochondrial damage and functional failure^5,6^. Mitochondria in neurons are the main source of energy and for any reason they become less functional in neurodegeneration and particularly in AD, resulting in energetic deficiency with the disease progression. It is not clear if this could be consequence of the pathological conditions and if could be an early event in the disease.

It is known that excessive production of Aβ peptide can be removed by an autophagy-dependent mechanism and this is confirmed by a number of studies^7–11^. Contrarily, it has been demonstrated that Aβ peptide accumulation can be an autophagy trigger itself so we can speculate that autophagy fails at one point in the disease progression and this could be consistent with the amyloid accumulation in the progression of AD. There are a number of studies showing that in addition of autophagy, Aβ peptide may induce mitophagy in AD. Mitophagy is a mitochondrial quality control that selectively removes damaged or superfluous mitochondria by autophagy^12^. This has been shown using cellular and animal models of the disease but it is not clear if this could be an early or late event in the pathology^13–18^.

Here we analysed the effect of Aβ peptide accumulation in the mitochondria of the most affected brain structures in AD, the hippocampus and cerebral cortex from 3, 6 and 12 month-old APP/PS1 mice. We noticed that mitochondrial mass was reduced in both regions from APP/PS1 mice before such event occurs in wild-type (wt) mice. This could be explained as the mitochondrial biogenesis is reduced but also because we found early impairments in the mitochondrial dynamics that ultimately increases mitophagy. Additionally, we found that Aβ overload in APP/PS1 mice increased autophagy highlighting a dual effect of Aβ accumulation in AD. We confirm all these results related to Aβ overload using primary neuronal and SH-SY5Y cellular models. Our results suggest that Aβ overload exert a dual effect increasing mitophagy and autophagy early in the AD pathology and this is maintained in aging.

## Results

### Mitochondrial mass is reduced in APP/PS1 mice due to amyloidosis

**Figure 1:**
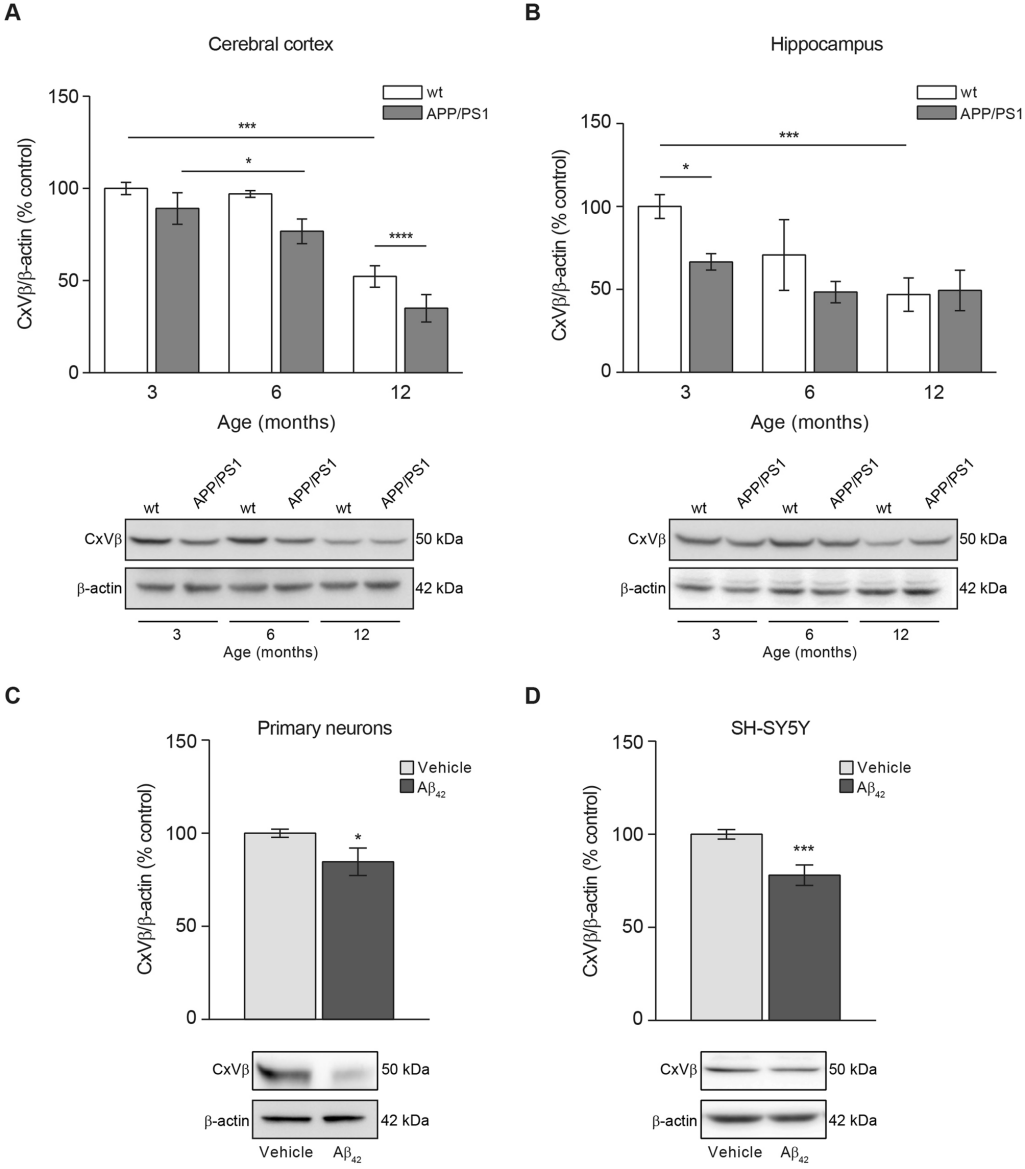
Aβ overload lowers the mitochondrial mass. Mitochondrial mass was studied by analysing the levels of the beta subunit of mitochondrial Complex V (CxVβ) as structural protein. The levels of CxVβ in cerebral cortex (**A**) and hippocampus (**B**) from 3-, 6- and 12-month-old wt and APP/PS1 mice are shown. Upper panels show histograms representing the protein densitometric analysis and representative experiments are presented in the bottom panels. Data are expressed as mean ± SEM; n = 5–10 mice. *p < 0.05; ***p < 0.001. Statistical significance was assessed by two-way ANOVA followed by Tukey’s post hoc test for multiple comparisons. CxVβ levels in rat primary neurons (C) and SH-SY5Y cells (D) treated with 1 µM oligomerised Aβ_42_ for 24 h. Upper panels show the protein levels estimation and representative immunoblots are reflected at the bottom. Data are expressed as mean ± SEM; primary neurons: n = 7, SH-SY5Y cells: n = 6. *p < 0.05; ***p < 0.001. Statistical significance was assessed by student’s t-test.

Aβ accumulation and overload may have effects on the mitochondrial health and function with consequences in processes that controls mitochondrial dynamics balance. Both, increased accumulation of defective mitochondria and excessive elimination of functional mitochondria may have detrimental effects for cells. For this reason, we determined whether the amount of mitochondria was altered due to Aβ overload in AD progression, using the APP/PS1 mouse model. We verified the Aβ accumulation in cerebral cortex and hippocampus of APP/PS1 mice, by ELISA (Supplementary Fig. 1). Aβ_40_ levels in cerebral cortex and hippocampus from 12-month-old mice were found significantly increased compared with 3- (p < 0.0001 in cortex; p < 0.0001 in hippocampus) and 6-month-old (p < 0.001 in cortex; p < 0.0001 in hippocampus) old mice (Supplementary Fig. 1A,B). Equivalent results were obtained regarding the Aβ_42_ levels (p < 0.001 comparing 12- with 3-month-old mice and p < 0,01 comparing 12- with 6-month-old mice in cortex; p < 0.05 comparing 12- with 3-month-old mice in hippocampus; Supplementary Fig. 1C,D). These result suggested the age-dependent accumulation of Aβ peptide. The amount of mitochondria was estimated by analysing the protein levels of CxVβ, a mitochondrial structural protein that may account the mitochondrial mass in cortical and hippocampal tissues from APP/PS1 and wt mice at 3-, 6-, and 12-month-old ages. Mitochondrial mass significantly decreased in APP/PS1 compared with wt mice (Fig. 1A,B). Such decrease was detected in the 3 month-old mice hippocampus (Fig. 1B) and later in the cerebral cortex (Fig. 1A). The reduced mitochondrial mass was maintained with age in the cerebral cortex but it is worth to mention that wt 12-month old mice also showed a mitochondrial mass reduction in this tissue but still this reduction was not that significant as showed by the 12 month-old APP/PS1 mice (Fig. 1A). In the hippocampus, the mitochondrial mass was reduced in both, wt and APP/PS1 12-month-old mice, equally (Fig. 1B).

The reduction of the mitochondrial mass due to Aβ accumulation was confirmed in rat primary neuronal cell cultures and SH-SY5Y cells after 24 h incubation with oligomerised Aβ_42_ (Fig. 1C,D). Both, rat primary neuronal cell cultures and SH-SY5Y cells showed a reduction in the CxVβ levels by 15% and 22% compared with untreated cells, respectively (Fig. 1C,D).

Our results indicate that mitochondrial mass reduction due to Aβ overload can be considered an early event in the AD development as demonstrates APP/PS1 animal model.

### Mitochondrial biogenesis is impaired in APP/PS1 mice

**Figure 2:**
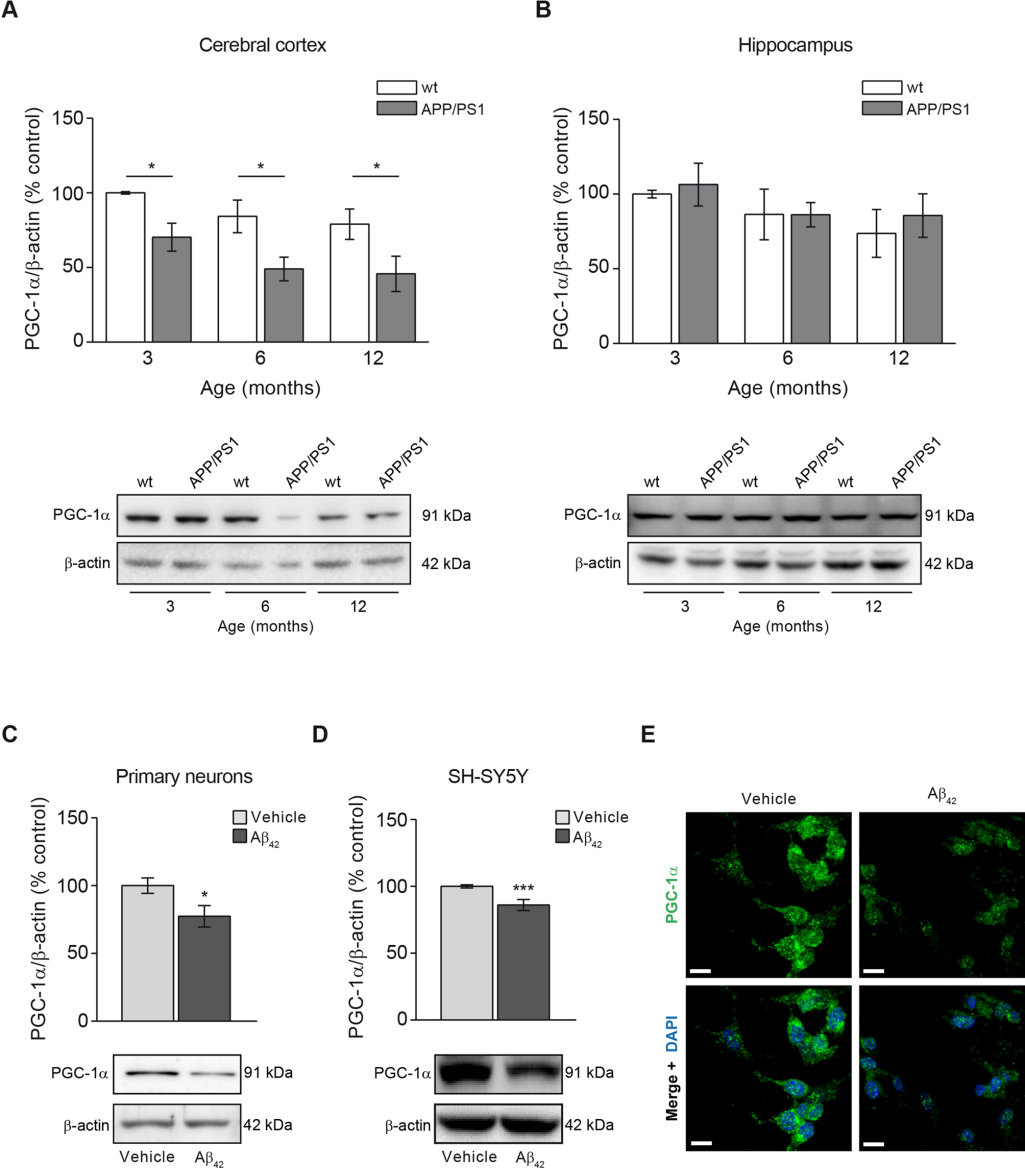
The mitochondrial biogenesis indicator PGC-1α is reduced due to Aβ overload. Mitochondrial biogenesis was studied by analysing the levels of the mitochondrial biogenesis master regulator, the transcription factor PGC-1α. The levels of PGC-1α in cerebral cortex (**A**) and hippocampus (**B**) from 3-, 6- and 12-month-old wt and APP/PS1 mice are shown. Upper panels show histograms representing the protein densitometric analysis and the bottom panels present representative experiments. Data are expressed as mean ± SEM; n = 5–10 mice. *p < 0.05. Statistical significance was assessed by two-way ANOVA followed by Tukey’s post hoc test for multiple comparisons. Histograms showing the PGC-1α levels in rat primary neurons (**C**) and SH-SY5Y cells (**D**) treated with 1 µM oligomerised Aβ_42_ for 24 h. Representative images of immunoblots are presented at the bottom panels. Data are expressed as mean ± SEM; primary neurons: n = 7, SH-SY5Y cells: n = 6. *p < 0.05; ***p < 0.001. Statistical significance was assessed by student’s t-test. (**E**) Immunofluorescence showing PGC-1α of SH-SY5Y cells treated with and without 1 µM oligomerised Aβ_42_ for 24 h. Cytoplasmic distribution of PGC-1α in cells is shown in green and the nuclei stained with DAPI are shown in blue. Scale bar = 14 µm.

A reduction in the mitochondrial mass could be consequence of a diminished mitochondrial biogenesis rate. We then analysed the levels of PGC-1α, the master regulator of mitochondrial biogenesis^19^. The analysis of this marker in the different animal groups showed that the reduction of the mitochondrial biogenesis was an early event in the cerebral cortex from APP/PS1 when compared with wt mice as the PGC-1α marker was found significantly reduced at 3-month-old and such levels were maintained with aging (3 months, p < 0.05; 6 months p < 0.05; 12 months, p < 0.05; Fig. 2A). PGC-1α levels in wt mice cerebral cortex did not change at any age (Fig. 2A). In the hippocampus, however no significant changes in PGC-1α levels were observed in any of the animal groups tested over age (Fig. 2B).

The reduction of PGC-1α levels due to Aβ overload was also confirmed in vitro where we found a significant reduction of this marker in rat primary neuronal cell cultures and SHSY5Y cells after incubation with oligomerised Aβ_42_ for 24 h (23% and 15% reduction compared with untreated cells, respectively; Fig. 2C–E). These results were supported by the analysis of the mitochondrial transcription factor A (TFAM) in SH-SY5Ycells and rat primary neurons both treated with oligomerised Aβ_42_ for 24 h (Supplementary Fig. 2). We could observe that Aβ_42_ treatment significantly diminished TFAM levels in SH-SY5Y cells and was observed as a trend-to-reduction in rat primary neurons (Supplementary Fig. 2).

### Aβ overload induces imbalances in the mitochondrial fusion and fission events

**Figure 3:**
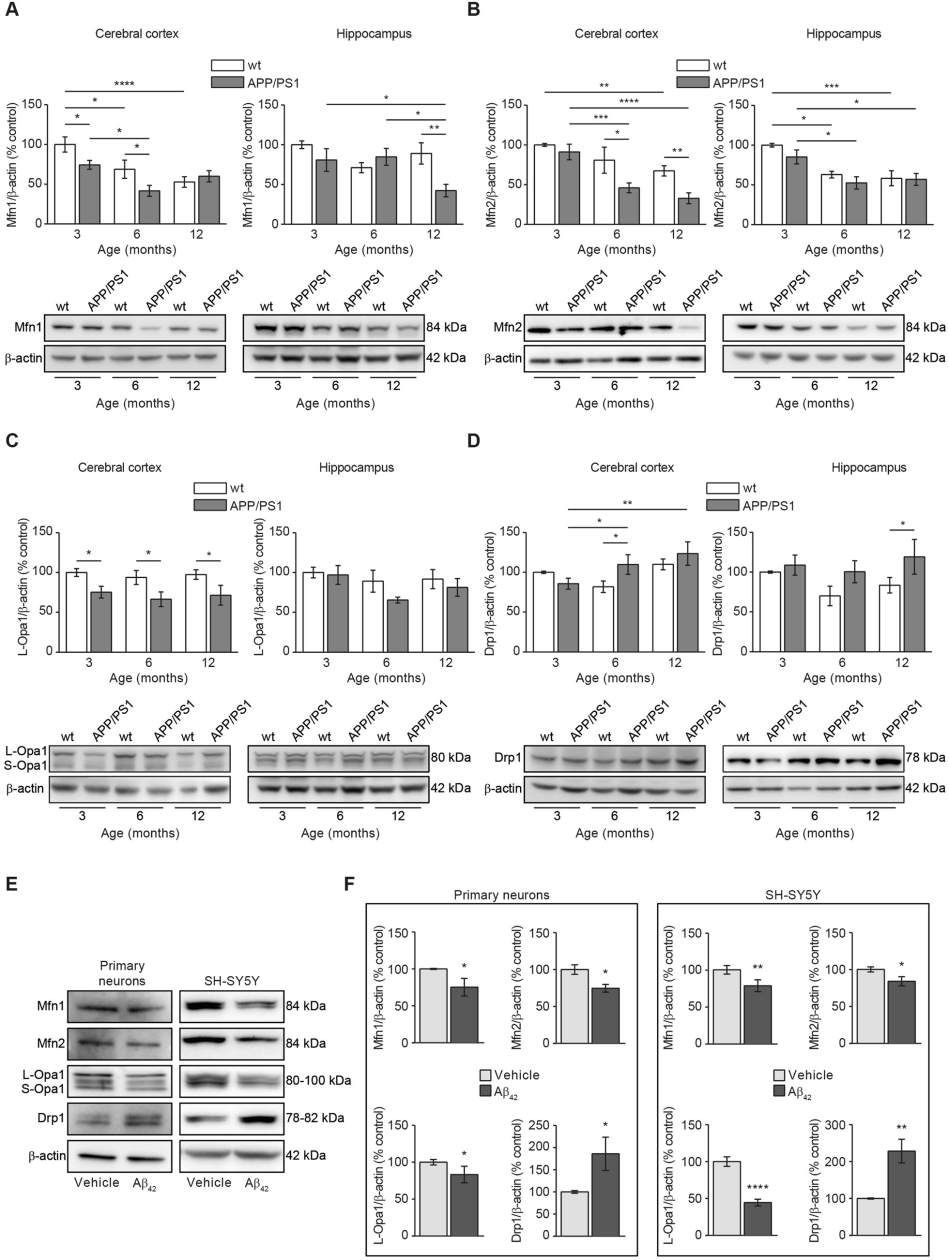
Aβ overload induces alterations in the mitochondrial dynamics towards increasing fission and reducing fusion balance. Mitochondrial dynamics was evaluated by analysing the levels of representative mitochondrial Mfn1 (**A**), Mfn2 (**B**) and Opa1 (**C**) fusion and Drp1 (**C**) fission proteins. In all cases (**A**–**D**) Histograms represent the densitometric protein levels in cerebral cortex (left panels) and hippocampus (right panel) from 3-, 6- and 12-month-old wt and APP/PS1 mice. Bottom panels show representative immunoblots. Data are expressed as mean ± SEM; n = 5–10 mice. *p < 0.05; **p < 0.01; ***p < 0.001; ****p < 0.0001. Statistical significance was assessed by two-way ANOVA followed by Tukey’s post hoc test for multiple comparisons. (**E**) Representative immunoblots showing the mitochondrial fusion (Mfn1, Mfn2 and Opa1) and fission (Drp1) proteins in rat primary neuronal cell cultures and SH-SY5Y cells after 24 h treatment with 1 µM oligomerised Aβ_42_. (**F**) Histograms presenting the densitometric analysis of the mitochondrial fusion (Mfn1, Mfn2 and Opa1) and fission (Drp1) proteins in rat primary neuronal cell cultures (left panel) and SH-SY5Y cells (right panel) after 24 h treatment with or without 1 µM oligomerised Aβ_42_. Data are expressed as mean ± SEM; primary neurons: n = 7, SH-SY5Y cells: n = 6. *p < 0.05; **p < 0.01; ****p < 0.0001. Statistical significance was assessed by student’s t-test.

A second explanation related to the reduced mitochondrial mass found in our Aβ overload models could be an increased mitochondrial degradation. Healthy mitochondria are fused composing a network thanks to the balance of fusion and fission mitochondrial proteins^20^. Upon mitochondrial damage, such balance is disrupted because levels of fusion proteins become reduced and levels of fission proteins become increased pushing the damaged mitochondria to split from the healthy network^20^. We analysed the levels of the mitochondrial fusion proteins Mfn1, Mfn2, Opa1, and those from the mitochondrial fission protein Drp1 in brain tissue from APP/PS1 and wt mice at different ages (Fig. 3A–D). Mfn1 levels were significantly reduced in cerebral cortex from APP/PS1 mice compared with wt mice in an early age (3-month-old) and such reduction was maintained as the mice over age. It is worth to mention that a significant reduction in Mfn1 levels was observed also in 6-month-old wt mice compared with 3-month-old animals from the same genotype, indicating a possible age-dependent feature (p < 0.05; Fig. 3A). In the hippocampus, a significant reduction in the Mfn1 levels was only observed in 12 month-old APP/PS1 mice compared with the age-matched wt mice (p < 0.01; Fig. 3A).

Levels of Mfn2 in the cerebral cortex showed a similar trend to that previously observed with Mfn1 levels, but its reduction in APP/PS1 mice came out later (6-month-old mice) (Fig. 3B). We found a decrease in Mfn2 levels in 6-, and 12-month-old APP/PS1 mice compared to wt mice groups with the same age (6 month-old, p < 0.001; 12 month-old, p < 0.0001; Fig. 3B). In wt mice, such decrease in Mfn2 levels was significantly evident later (12 month-old; p < 0.01; Fig. 3B). In the hippocampus, Mfn2 levels were significantly reduced in 6- and 12-month-old wt (p < 0.05; p < 0.001; Fig. 3B) and APP/PS1 mice (p < 0.05; Fig. 3B), indicating that such reduction could be consequence of aging.

Regarding the inner mitochondrial membrane fusion protein Opa1, we found a significant reduction in APP/PS1 cerebral cortex levels early in age (3 month-old) compared with the age-matched wt mice and such significance was maintained as mice with aging (p < 0.05; Fig. 3C). Wt mice did not show reduction in Opa1 levels with age at cortical level (Fig. 3C). In the hippocampus, no significant reduction was observed in Opa1 levels in both APP/PS1 and wt mice (Fig. 3C).

In summary, our data suggest that mitochondrial fusion could be compromised in cerebral cortex from APP/PS1 mice as mitochondrial fusion protein levels were found reduced compared with those levels from wt animals, and could be considered an early detectable event. In hippocampus, such effect would be only residual as only the levels of Mfn1 fusion protein were found reduced in 12-month-old APP/PS1 mice, and Mfn2 reduction could be an age-dependent effect as it was evident in both, wt and APP/PS1 mice.

In contrast to fusion proteins, Drp-1 levels were significantly increased in the cerebral cortex from 6- and 12-month-old APP/PS1 mice compared with 3 month-old APP/PS1 and wt mice with the same age (p < 0.01; Fig. 3D). Again, these results point to an increased mitochondrial fission rate in the APP/PS1 cerebral cortex. No statistical differences were detected at hippocampal level (Fig. 3D).

To address if such fusion-fission imbalance is consequence of amyloidosis, we analysed the levels of the same proteins in our in vitro models, rat primary neuronal cell cultures and SH-SY5Y cells, 24 h after incubation with Aβ_42_. As shown in Fig. 3E,F, in both cellular models, Aβ induced a significant reduction in the fusion protein levels Mfn1, Mfn2 and Opa1, and a significant increase in the fission protein levels Drp1 (Primary neurons: Mfn1 = 25%, Mfn2 = 25% and Opa1 = 23% reduction, and Drp1 = 86% increase compared with untreated cells; SH-SY5Y cells: Mfn1 = 21%, Mfn2 = 16%, Opa1 = 55% reduction, and Drp1 = 128% increase compared with untreated cells; Fig. 3E,F). These results confirm that Aβ overload may induce an imbalance in the fusion-fission proteins equilibrium that ultimately may drive the damaged mitochondria to mitophagy.

### Aβ overload triggers mitophagy

**Figure 4:**
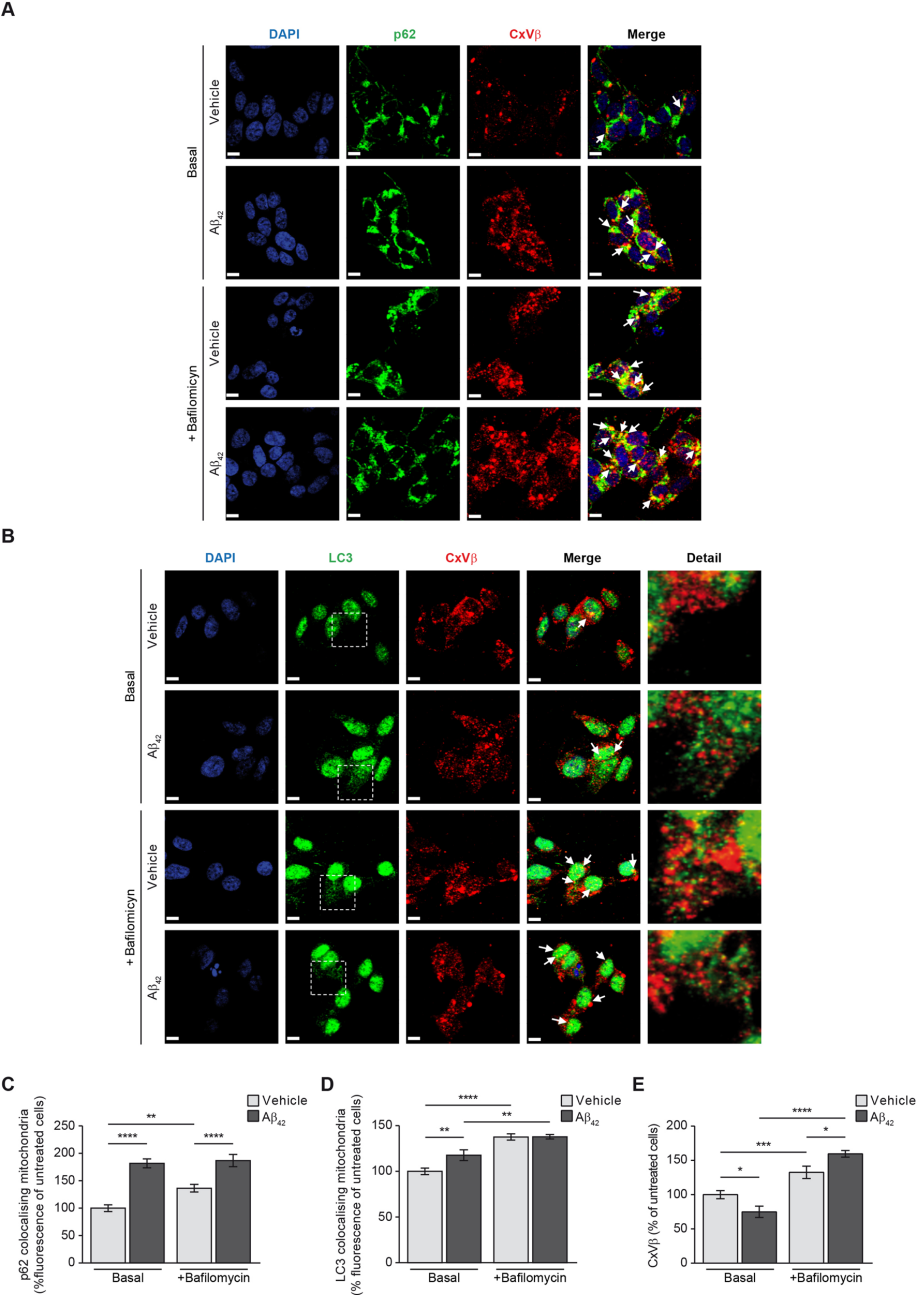
Aβ increases mitophagy. The effect of Aβ on mitophagy was evaluated in SH-SY5Y cells by analysing the colocalisation of the mitochondria with the main autophagy markers p62 (**A** and **C**) and the punctate-like structures of LC3-II (**B** and **D**) by immunofluorescence (white arrowheads). Representative images of SH-SY5Y cells treated with or without 1 µM oligomerised Aβ_42_ for 24 h (Basal). The autophagosome containing mitochondria accumulation was evaluated by addition of the autophagosome—lysosome fusion inhibitor bafilomycin (100 nM). In all cases, the mitochondria were located using the specific antibody against the structural protein beta subunit of mitochondrial Complex V (CxVβ; stained in red). The p62 (**A**) and punctate-like structures of LC3-II (**B**) as autophagy markers were located using the specific antibodies (stained in green) and the nuclei were stained with DAPI (blue). Scale bar = 9 µm. (**C** and **D**) Histograms show only the colocalisation levels (yellow signal in the images, and arrowheads) between the mitochondria and p62 (**C**) and between the mitochondria and LC3-II (D) that were estimated analysing the fluorescence in the basal stage and in the presence of bafilomycin (100 nM) with or without 1 µM oligomerised Aβ_42_ for 24 h. (**E**) Histogram showing the amount of mitochondria that was estimated by analysing the fluorescence levels of the structural protein CxVβ with or without 1 µM oligomerised Aβ_42_ for 24 h and in absence (Basal) or presence of bafilomycin (100 nM). Data are expressed as mean ± SEM; n = 5. *p < 0.05; **p < 0.01; ***p < 0.001; ****p < 0.0001. Statistical significance was assessed by one-way ANOVA followed by Fisher’s post hoc test for multiple comparisons.

We next analysed in our cellular models if amyloid overload may be responsible of increased mitophagy by analysing the colocalisation of cytosolic autophagy markers p62 and LC3 with the mitochondria only. This would reflect the amount of mitochondria prompted to mitophagy. The analysis was carried out in SH-SY5Y cells incubated with or without oligomerised Aβ_42_ for 24 h (basal stage) and in absence or presence of the specific vacuolar H^+^ ATPase (V-ATPase) inhibitor bafilomycin (100 nM). Bafilomycin prevents the maturation of autophagic vacuoles by inhibiting late-stage fusion between autophagosomes and lysosomes as well as lysosomal degradation^21^. Due to its ability to specifically target V-ATPase, bafilomycin disrupts autophagic flux, and is frequently used to study autophagy and endosomal acidification. Then we used bafilomycin to analyse the accumulation of autophagosome-engulfed mitochondria. Figure 4A,B shows representative images of the mitophagy markers p62 (Fig. 4A) and LC3-II (Fig. 4B) colocalising with the structural mitochondrial protein beta subunit of Complex V (CxVβ) in absence or presence of bafilomycin. The amount of colocalised mitochondria with cytosolic p62 and punctate LC3-II was estimated using the Volocity software as it is indicated in the “Methods” Section (Fig. 4C,D). The analysis showed that Aβ_42_ incubation increased mitophagy as demonstrated the increase of autophagy markers colocalising with the mitochondria (Manders’ coefficients for p62 and CxVβ: untreated cells, M1 = 1, M2 = 0.89; cells with Aβ_42_, M1 = 1, M2 = 0.97; Manders’ coefficients for LC3-II and CxVβ: untreated cells, M1 = 1, M2 = 0.89; cells with Aβ_42_, M1 = 1, M2 = 0.96; Fig. 4C,D). The effect of Aβ_42_ increasing mitophagy was confirmed by using bafilomycin as this compound showed the maximal autophagosome-engulfed mitochondria accumulation in Aβ_42_-treated SH-SY5Y cells (Manders’ coefficients for p62 and CxVβ: cells with bafilomycin, M1 = 1, M2 = 0.92; cells with Aβ_42_ and bafilomycin, M1 = 1, M2 = 0.96; Manders’ coefficients for LC3 and CxVβ: cells with bafilomycin, M1 = 1, M2 = 0.95; cells with Aβ_42_ and bafilomycin, M1 = 1, M2 = 0.96; Fig. 4C,D). Additionally, the analysis also probed that Aβ_42_ overload reduced the amount of mitochondria (Fig. 4E) consistent with the previous results (Fig. 1D). The presence of bafilomycin in Aβ_42_-treated SH-SY5Y cells showed increased levels of the mitochondrial marker CxVβ, possibly indicating that the autofagosome-containing mitochondria did not fuse with the lysosomes (Fig. 4E).

### Aβ overload increases autophagy

**Figure 5:**
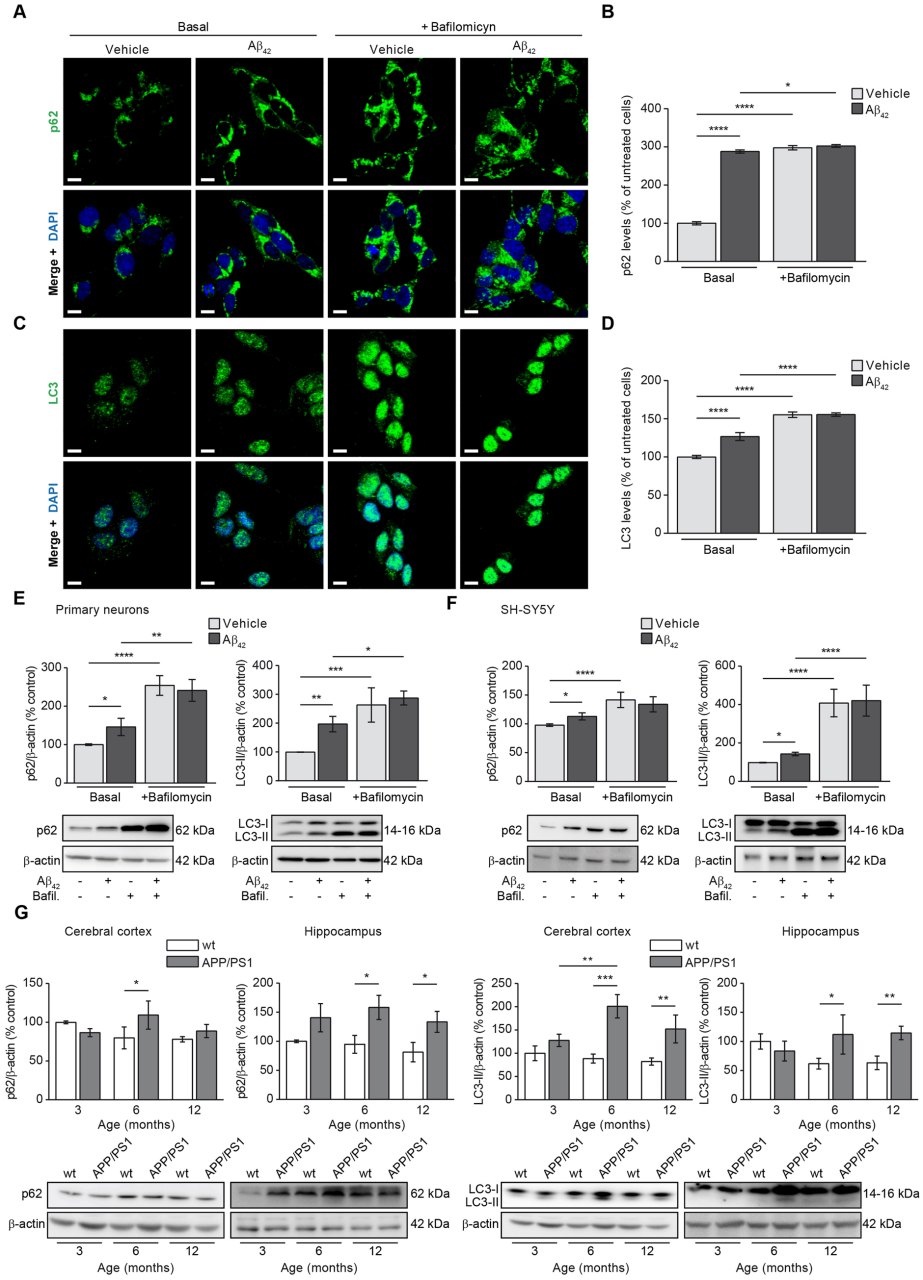
Autophagy is increased upon Aβ overload. The effect of Aβ on autophagy was evaluated in SH-SY5Y cells by analysing the levels of the main autophagy markers p62 (**A** and **B**) and LC3 (**C** and **D**) by immunofluorescence. Representative images of SH-SY5Y cells treated with or without 1 µM oligomerised Aβ_42_ for 24 h (Basal) showing the autophagy marker p62 (**A**) and punctate-like structures of LC3-II (**C**) in green. The autophagosome accumulation was evaluated by addition of the autophagosome—lysosome fusion inhibitor bafilomycin (100 nM). In all cases the nuclei were stained with DAPI (blue). Scale bar = 9 µm. Histograms show the levels of p62 (**B**) and LC3-II (**D**) that were estimated analysing the fluorescence in the basal stage and in the presence of bafilomycin (100 nM) with or without 1 µM oligomerised Aβ_42_ for 24 h. (**E** and **F**). Data are expressed as mean ± SEM; n = 5. Statistical significance was assessed by one-way ANOVA followed by Fisher’s post hoc test for multiple comparisons. *p < 0.05; ****p < 0.0001. Autophagy levels were verified analysing the p62 and LC3-II levels by immunoblotting in rat primary neuronal cell culture (**E**) and SH-SY5Y cells (**F**) with or without 1 µM oligomerised Aβ_42_ for 24 h in absence (Basal) or presence of bafilomycin (100 nM). In all cases top panels show the histograms indicating the protein levels estimation for each marker and the bottom panels show representative western blots. Data are expressed as mean ± SEM; primary neurons: n = 4, SH-SY5Y cells: n = 7. Statistical significance was assessed by one-way ANOVA followed by Fisher’s post hoc test for multiple comparisons. *p < 0.05; **p < 0.01; ***p < 0.001; ****p < 0.0001. (**G**) Autophagy was evaluated in mice by analysing the levels of representative autophagy markers p62 (panel first and second from left to right) and LC3-II (panel third and forth from left to right). In all cases, histograms represent the densitometric protein levels in cerebral cortex (panel first and third from left to right) and hippocampus (panel second and fourth from left to right) from 3-, 6- and 12-month-old wt and APP/PS1 mice. Bottom panels show representative immunoblots. Data are expressed as mean ± SEM; n = 5–10 mice. *p < 0.05; **p < 0.01; ***p < 0.001. Statistical significance was assessed by two-way ANOVA followed by Tukey’s post hoc test for multiple comparisons.

Imunufluorescence showing the colocalisation of cytosolic autophagy markers with the mitochondria revealed not only increased mitophagy due to Aβ_42_ overload but also increased levels of autophagy markers in general. We then verified by immunofluorescence as it is specified in the “Methods” section the levels of p62 and LC3-II in SH-SY5Y cells to know the effect of Aβ_42_ in autophagy. Figure 5A,C shows representative images of p62 and LC3-II fluorescence in cells incubated with or without oligomerised Aβ_42_. Both, p62 and punctate LC3-II fluorescence levels were found increased in the Basal stage (without bafilomycin) upon amyloid incubation and bafilomycin increased the autophagosome accumulation in cells but much more under Aβ_42_ treatment condition (Fig. 5B,D). Such results were validated by immunoblotting in rat primary neuronal cell cultures (Fig. 5E) and SH-SY5Y cells (Fig. 5F). The above results showed that Aβ overload in addition to increase mitophagy it may increase also autophagy. It is possible that such autophagy increase could be related to the described higher autophagy flux in AD patients as an elimination mechanism of the peptide in early stages of the disease. To know about such possible relationship, we analysed in vivo how the autophagy markers changed along with the mice age in brain tissue from APP/PS1 mice compared with those from the wt. Figure 5G show how p62 and LC3-II levels were significantly increased in both, cerebral cortex and hippocampus from 6-month-old APP/PS1 mice but not in the wt (Fig. 5G). With the exception of p62 marker in cerebral cortex, such increase was maintained in the 12-month-old animals (Fig. 5G).

Altogether the results in mice are consistent with those obtained in cell cultures where we found that Aβ incubation had a dual effect: increasing mitophagy and autophagy. Our results in mice suggest that Aβ overload may induce autophagy at early ages and this is maintained in elderly which would be equivalent to the observed increased autophagy in patients at early stages of AD according to amyloidosis.

## Discussion

In this work, we have analysed the effects of amyloid overload on mitochondrial dynamics, mitophagy and autophagy in APP/PS1 mice from 3- to 6- and 12-month-old age. We have found that Aβ accumulation diminishes the mitochondrial mass due to a reduction in the mitochondrial biogenesis and increased mitophagy. This was an early event in the APP/PS1 mice hippocampus (3-month-old) compared with wt mice group in which the reduction in the mitochondrial mass was evident at 12-month-old age. Imbalances in the mitochondrial fusion and fission proteins were found biased towards mitochondrial fission and therefore, to mitophagy. We confirmed the effect of Aβ overload by using rat primary neuronal cell cultures and SH-SY5Y cells subjected to oligomeric Aβ_42_ treatment, as they also showed reduced mitochondrial mass and biogenesis, altered mitochondrial dynamics prompted to mitochondrial fission, and increased mitophagy. In addition to mitophagy, we observed that Aβ overload induced a dual effect by increasing the autophagy from early stages of the amyloidogenic process in APP/PS1 mice and in all cellular models.

Here, we found that most of the proteins studied were found altered earlier in the brain cortex from APP/PS1 mice than in the hippocampus (mitochondrial mass, Mfn1, Mfn2, and Drp1) or even remained unaltered (PGC-1a and Opa1). This could be related with the lower Aβ levels found in the hippocampus from APP/PS1 mice compared with those in the cortex. The increased autophagy activity found in the hippocampus of APP/PS1 mice from 6-month-old onwards might be enough to remove the expected increased amyloid peptide levels in this region. This would avoid the Aβ effects on the mitochondrial dynamics in the hippocampus. A novel explanation linking the damaged mitochondrial activity could be related to the effect of increased APP-derived fragments other than Aβ as was shown in different works from Area-Gomez group^22–24^.

Defective mitochondrial dynamic balance has been suggested to be one of the reasons as well as consequence of AD-related pathology. Autophagy in neurons is important under physiological and pathological conditions playing a crucial role for the degradation of Aβ. Although this could represent a controversial issue, a recent work established an interlink mechanism for the mitochondrial dysfunctioning, oxidative stress, autophagy dysregulation, and neuronal cell death in AD^25^. The reasons by which AD pathology occurs are still far to be elucidated, and a huge subset of factors may contribute to the origin of the disease. Such factors have built additional hypothesis other than amyloid cascade that may explain the origin of AD.

A reduction in the mitochondrial mass is recurrent in neurodegeneration. Neurons may die because energetic collapse due to the lack of power production which is carried out by the mitochondria. Related to AD, it was found a reduction in the amount and volume of mitochondria along with increased mitochondrial fragmentation and impaired mitochondrial transport in Aβ-treated mouse hippocampal neurons^26^. Reduced mitochondrial mass was also found in brain lysates and hippocampal sections from AD patients^27–29^. Consistent with the mentioned studies, we demonstrate here that mitochondrial mass reduction in AD could be consequence of Aβ accumulation and also we probe that in general this would be an early effect in the progression of disease. However it is worth to mention that in the hippocampus the loss of mitochondrial mass could be consequence of other additional disease-triggering events or undetectable but still pathological levels of Aβ as we already found such reduction in the 3-month old mice along with no evidence of amyloid overload.

Low amount of mitochondria in brain of APP/PS1 mice may reflect in some cases was found to be consequence of reduced mitochondrial biogenesis as we found in the present study. Mitochondrial biogenesis is controlled by several transcription factors and PGC-1α is the main regulator^30^. Our results in the cerebral cortex of APP/PS1 mice showing reduced PGC-1α levels at early age accounted of the reduced mitochondrial mass found in that brain region. This was consistent with the reduced PGC-1α levels and mRNA expression found in other previous studies^31–33^. In brains from AD patients, a relationship between reduced PGC-1α levels and damaged mitochondrial biogenesis was also previously demonstrated^34,35^. Other works also demonstrated that the reduction in the PGC-1α levels were consequence of Aβ overload using similar cellular models as in our present study^17,36,37^. In those cases authors also showed the link with reduced mitochondrial biogenesis. Hippocampal analysis of PGC-1α however did not account the reduced mitochondrial mass found in this region. Also we would like to mention that reduced PGC-1α levels in brain cortex form APP/PS1 mice were evident at 3-month old mice but the mitochondrial mass was reduced at 6-month old mice. For this reasons, we also searched other possible causes conditioning the mitochondrial mass that could add up to the reduction in case of the hippocampus or exert a compensatory mechanism in case of the brain cortex at early ages in the APP/PS1 mice brains. This could be the increased mitochondrial degradation by mitophagy, which is preceded by imbalances in the mitochondrial fusion/fission proteins such as Mfn1, Mfn2, Opa1 and Drp1 among others. We found a reduction in the levels of fusion proteins Mfn1, Mfn2 and L-Opa1 at early ages in brain regions from APP/PS1 mice. Contrarily, the levels of the fission protein Drp1 were found increased but this observation was not found as early as the reduction in the fusion protein levels. This could be explained because higher levels of amyloid accumulation are necessary to induce effects on Drp1 levels or because this fission protein could exert an early stage compensatory effect avoiding the mitochondrial degradation. This is consistent with the unchanged mitochondrial mass levels in the brain cortex from 3-month-old APP/PS1 mice where a reduction of the CxVβ levels was found from 6-month-old mice onwards. Again, our experiments in cells incubated with oligomerised Aβ confirmed that such effects were due to amyloid overload. Therefore we may conclude that in our AD mouse model, a disequilibrium between the fusion and fission processes exists and this may mimic AD pathology encouraging the mitochondrial fission and then mitophagy.

Studies carried out in primary neurons described similar effects on mitochondrial fission and fusion events, in which the treatment with diffuse forms of the Aβ peptide showed a decrease in the levels of mitochondrial fusion proteins (Mfn1, Mfn2 and Opa1)^27,38^ and an increase in the levels of mitochondrial fission proteins (Drp1)^39^. Also other AD transgenic mice exhibited increased mitochondrial fission processes in their brains^40–42^. In neuronal cell cultures from AD mouse models, authors demonstrated these imbalance encouraging the mitochondrial fission were demonstrated with increased fission and reduced fusion mRNA expression and protein levels^26^. Consequence of these alterations, mitochondrial morphology was found modified as they display small and rounded shape, and increased fragmentation with possible negative consequences in the mitochondrial function. The imbalance of the mitochondrial fusion and fission proteins in AD was previously proposed as an early event of the disease progression^27,43^, and these were supported by evidences of changes in the fusion and fission protein levels towards mitochondrial fission in brain tissue from AD patients^27,44^.

The fission of damaged mitochondria leads to their degradation by mitophagy^45^. We verified the increase in mitophagy in SH-SY5Ys cells after incubation with Aβ, obtaining increased colocalisation of autophagy markers p62 and LC3 with mitochondria, as well as mitochondrial accumulation after applying bafilomycin. Some works agree with our observations, also indicating that the Aβ peptide may be a trigger for mitophagy^13–18,46^. This was suggested by studies in which it was shown in mouse primary neuronal cell cultures that exposure to oligomeric Aβ_42_ induced imbalances in the transcription of genes encoding mitochondrial fission and fusion proteins, increasing mitochondrial fission and autophagy^47,48^.

Our results show a dual effect of Aβ peptide overload. Aβ exerts a detrimental effect on mitochondria, possibly compromising their function and processes such as calcium homeostasis, ATP generation, and apoptosis. On the other hand, Aβ overload increased autophagic flux, as we have verified in our in vitro but also in our in vivo AD mouse models in which an increase in autophagic markers p62 and LC3-II was observed. There is some controversy regarding the interpretation of the levels of p62. Some authors have shown a decrease in the levels of this autophagy marker in brains from AD patients^49,50^ while other works reported an increase^46,51^. It is possible that these variations within the same pathological conditions in p62 levels are related to the involvement of this protein in other cellular processes as p62 by interaction through its different functional domains or by variation experimental when analysing the levels of this protein in each of these works^52,53^.

We have seen an increase of LC3-II upon oligomeric Aβ_42_ administration in SH-SY5Ys cells. The administration of bafilomycin in the cell cultures caused the levels of both p62 and LC3-II to increase, more significantly in the presence of Aβ_42_ indicating higher autophagy flux. Alterations in autophagy have been seen in in vitro and in vivo models of AD, and by analysis of postmortem AD brains^54^. Increased LC3-II levels have been found in the cerebral cortex and hippocampus of APP/PS1 mice at different ages, suggesting autophagosome accumulation in AD^55–57^. In addition to protein levels^11^, other authors have shown that LC3-II and p62 mRNA levels also increased after treatment with Aβ in vitro^9^.

Taken together, our results show that the Aβ peptide produces a dual effect as we have shown in our in vitro experiments. This dual effect could be extrapolated to in vivo as we have seen that the autophagy process is exacerbated in APP/PS1 mice at early age and the mitochondrial dynamics imbalances also occur in these stages. Mitochondrial biogenesis and fusion/fission protein levels became altered with advanced age but these features occurred much late compared with the APP/PS1 mice, observation that could be attributable to aging as risk factor affecting mitochondrial activity. Although it has not been corroborated at the mitochondrial level, it is possible that these imbalances in the mitochondrial fusion and fission proteins end up triggering the elimination of damaged mitochondria and, therefore, confirming the double effect of the Aβ peptide in our in vivo model. It should be noted that, at older ages, despite the increase in autophagy, a greater accumulation of the Aβ peptide is observed, suggesting that, despite being able to be eliminated by autophagy, the production of Aβ would exceed elimination levels. This offers the possibility of studying in patients how the dual effect of Aβ can contribute both to its accumulation and to a reduction in energy production, both characteristic features of AD.

## Material and methods

To analyse the effects of Aβ on the mitochondria along with the progression of AD, brain regions of 3-, 6- and 12-month old APP/PS1 mice as Alzheimer’s disease mouse model were obtained and analysed as well as the same regions from wild type mice with equivalent ages. In these models, the levels of Aβ isoforms (Aβ_40_ and Aβ_42_) were confirmed along with time. Both Aβ isoforms result from the amyloidogenic processing of APP and are considered pathogenic. In the brain regions, protein levels of those related to mitochondrial biogenesis, dynamics and autophagy were analysed using biochemistry approaches. To confirm the role of Aβ in the observed alterations in brain tissue, the obtained results were also confirmed and studied in the human dopaminergic neuroblastoma cell line SH-SY5Y and neuronal primary cell cultures, both under incubation with oligomerised Aβ_42_ as this isoform is declared as the most toxic. In addition, the effect of Aβ on mitophagy was studied in the cell cultures analysing the levels and mitochondrial localisation of the main autophagy markers with the mitochondria. The biochemistry approaches utilised in cells were equivalent to those used in the mice brain tissue but also included immunofluorescence approaches. Below, the reagents, antibodies, animals, cell cultures and techniques utilised in this study are shown in detail.

### Reagents

Aβ_42_ stock was prepared by pouring the lyophilised monomeric Aβ_42_ in acetic acid 0.1 M. Prior to addition to the cell cultures, oligomeric Aβ_42_ was prepared by incubating a volume of the stock solution in DMEM at 4 °C for 24 h as previously described^58^. The analysis of autophagosome accumulation was carried out by using the autophagosome-lysosome fusion inhibitor bafilomycin (100 nM; Sigma-Aldrich, St. Louis, USA).

### Antibodies

Primary antibodies used were as follows: mouse anti-complex V β subunit (CxVβ; for Western Blotting-WB-, 1:1000; for Immunocytochemistry-ICC-, 1:200; Abcam, Cambridge, UK), rabbit anti-peroxisome proliferator-activated receptor γ co-activator 1 α (PGC1-α; for WB, 1:200; for ICC, 1:200; Santa Cruz Biotechnologies, CA, USA), rabbit anti- mtTFA (TFAM; 1:1000, Abcam Cambridge, UK), mouse anti-Mitofusin1 (Mfn1; 1:1000; Abcam, Cambridge, UK), mouse anti-Mitofusin2 (Mfn2; 1:1000; Abcam, Cambridge, UK) mouse anti-mitochondrial dynamin-like GTPase (OPA1; 1:1000; Novus Biologicals, CO, USA), rabbit anti-Dynamin-related protein 1 (Drp1; 1:1000, Cell Signalling Technology, MA, USA) mouse anti-β actin HRP (1:25,000; Abcam, Cambridge, UK), rabbit anti-p62/SQSTM1 (for WB, 1:20,000; for ICC, 1:200; Abcam, Cambridge, UK), rabbit anti-LC3 (for WB, 1:1000; for ICC, 1:200; Novus Biologicals, CO, USA). Secondary antibodies used for WB were as follows: goat anti-rabbit HRP secondary antibody (1:5000; Thermo Fisher Scientific, MA, US), goat anti-mouse HRP secondary antibody (1:5000; Abcam, Cambridge, UK). Secondary antibodies used for ICC were as follows: anti-rabbit (Alexa Fluor, emission at 488 nm; 1:1000; Thermo Fisher Scientific, MA, US) or anti-mouse (Alexa Fluor, emission at 568 nm; 1:1000; Thermo Fisher Scientific, MA, US).

### Animals

3-, 6- and 12-month-old male double transgenic APP/PS1 mice were used from our inbred colony^59^ (Instituto de Investigacion Hospital 12 de Octubre and Centro de Biología Molecular Severo Ochoa). APP/PS1 mouse model comes from a cross between Tg2576 (overexpressing human APP695) and mutant PS1 (M146L). As wild-type (wt) controls we used age-matched mice not expressing the transgene. For the sacrifice animals were deeply anesthetised. Then, they were perfused transcardially with saline before organ extraction for biochemical analysis. Sample size was estimated for each condition using the SPSS statistics software, assuming a normal distribution in all cases. This sample size is necessary to minimise the number of animals obtaining significant differences between groups and reducing the possible intragroup variability with an established statistical power of 95% confidence. In all cases, variability or standard deviation lower than the mean along with less than 10% of losses (mice deaths) are expected.

For rat primary neuronal cell cultures, pregnant Wistar rats (3–5 months) were obtained from the inbred colony of the Hospital Doce de Octubre Research Institute, Madrid, Spain.

All animals were handled and cared for according to the Council Directive 2010/63/UE of 22 September 2010 and the revisited ARRIVE guidelines (2020).

### Cell cultures

Primary cortical neuronal cultures were prepared from rat embryos (E15–16) based on methods previously described^59^. 4 × 10^5^ cells per well were plated on poly-l-lysine coated coverslips in 6-well plates. Cells were maintained at 37 °C in a 5% CO2 humidified atmosphere in Neurobasal A medium (Thermo Fisher Scientific, MA, US) containing 2 mM l-glutamine, and 10% B27 Supplement (Thermo Fisher Scientific, MA, US). 9 days after cell culture preparation, cortical neurons were treated with or without oligomerised Aβ_42_ (1 μM). All live-cell imaging experiments were performed between d10 and d14 in culture. Human dopaminergic neuroblastoma cell line (SH-SY5Y) was purchased from the European Collection of Cell Cultures (Health Protection Agency, Salisbury, UK) and maintained as previously described^60^. Unless otherwise stated, SH-SY5Y cells were seeded at a density of 4 × 10^4^ cells/cm^2^ and maintained in Dulbecco's modified Eagle's medium (DMEM) supplemented with 10% (v/v) fetal bovine serum (FBS), 2 mM l-glutamine, and 1% (v/v) Penicillin/Streptomycin at 37 °C and 5% CO_2_. Cell cultures were incubated in fresh medium with or without oligomeric Aβ_42_ (1 μM).

### Immunoassay for Aβ peptides (ELISA)

Levels of Aβ_40_ Aβ_42_ in cerebral cortex and hippocampus from APP/PS1 and wild type mice were determined using the human specific Aβ_40_ and Aβ_42_ ELISA kits (KHB3481 and KHB3544 respectively; Invitrogen, CA, US), according to the manufacturer’s instructions. The obtained data were normalised to the protein content.

### Immunoblotting

Proteins were isolated from brain tissue or cell cultures by standard methods^58^. Briefly, protein content from brain tissue or cell lysates was obtained using the NP-40 lysis buffer (50 mM Tris/HCl buffer, pH 7.4 containing 2 mM EDTA, 0.2% Nonidet P-40, 1 mM PMSF, protease and phosphatase inhibitor cocktails—Roche, Basel, Switzerland) and maintained at − 80 °C. The amount of protein was estimated using the BCA method (Thermo Fisher Scientific, MA, US). 20 µg of protein were loaded in SDS-PAGE gradient gels (4–20%). The gels with the separated proteins were transferred onto polyvinylidene difluoride (PVDF) membranes (Millipore, MA, USA). Each specific protein was determined using its specific primary antibody. Then, the appropriate horseradish peroxidase-conjugated secondary antibodies was used to bind each specific primary antibody and an enhanced chemiluminescence reagent (ECL Clarity; Bio Rad, CA, USA) was used to reveal the immunocomplexes using the ImageQuant LAS 4000 system (GE Healthcare). Protein loading was monitored using a mouse monoclonal antibody against β-actin. Protein densitometric quantification was carried out using the Image Studio Lite 5.0 software (Li-COR Biosciences, NE, USA). β-actin levels were used to normalise each protein levels and they are expressed as percentage of the control group. Upon requirements, in some cases blot membranes were cut to carry out simultaneous antibody hybridisations. In other cases blot membranes were covered during the digital exposure to avoid the signal of previous antibody incubations. These two points are specified in the legends of each corresponding figure within the supplementary information where the full-length blots are shown.

### Immunocytochemistry

For immunocytochemistry (ICC) experiments, 6 × 10^4^ cells were plated onto sterilised coverslips in 24-well plates. 24 h later, (when cells reached 80% confluence approximately) cells were fixed with 4% paraformaldehyde in 0.1 M Dulbecco's phosphate buffered saline (DPBS, Thermo Fisher Scientific, MA, US) for 10 min at room temperature. Cells were then washed three times in DPBS, blocked and permeabilised at room temperature for 45 min in 10% horse serum in DPBS with 0.1% Triton-X100 (Sigma-Aldrich, St. Louis, USA). Primary antibodies were prepared in the blocking/permeabilising solution and cells were incubated at 4 °C overnight. Secondary antibodies were used to reveal the primary antibody staining. Coverslips were mounted onto slides using the ProLong Gold Antifade Mountant with DAPI (Thermo Fisher Scientific, MA, US). Images were acquired with a Zeiss LSM 710 confocal microscope and processed by using the Zen 2009 software. Colocalisation analysis was carried out by using the Volocity software (Quorum Technologies, CA) and it was calculated following the instructions reported by the Software. For p62 and LC3 colocalisation with the mitochondria a number of 6 independent coverslips were analysed and results are referred as % of untreated cells. For experiments showing the p62 or LC3 levels, a number of 8 independent coverslips were analysed. For LC3-II and p62 analysis of mitophagy (Fig. 4), only the colocalisation of these antibodies with the mitochondrial CxVβ antibody was quantified using the “excluding non-colocalising objects tool” from the Volocity software. For the LC3-II analysis in mitophagy (Fig. 4), only the punctate-like structures colocalising with the mitochondrial CxVβ antibody were analysed and the nuclear staining was excluded using the “excluding non-touching objects tool” from the Volocity software. For LC3-II analysis for autophagy experiments (Fig. 5), only the punctate-like structures were analysed and the nuclear staining was excluded using the “non-touching objects tool” from the Volocity software.

### Statistical analysis

In vivo and in vitro results are shown related to wt mice and untreated cells, respectively. All of them are expressed as the mean ± standard error of the mean (SEM) in percentage. For animal experiments, sample size estimation was carried out using the IBM SPSS Statistics software, Version 21.0. (Armonk, NY, USA). Statistical analysis and exponential curve fitting were performed using the GraphPad Prism 6.01 (GraphPad Software, La Jolla, CA, USA) software. Grubbs outlier filter was used for all data and Shapiro–Wilk normality test indicated that all results met normality criteria. For two-group comparisons, statistical analysis was carried out using Student’s t-test. For more than two-group comparisons, the one-way Analysis of variance (ANOVA) followed by Fisher’s post hoc test for multiple comparisons was used. For experiments carried out with animals at different ages, the two-way Analysis of variance (ANOVA) followed by Tukey’s correction test was used Statistical significance was set at p < 0.05.

### Ethics statement

All animals were handled and cared for according to the Council Directive 2010/63/UE of 22 September 2010 and the revisited ARRIVE guidelines (2020) and approved by the Autonomous University Ethics Committee for Animal Experimentation (license number: CEI 97-1778-A291).

### Supplementary Information

Supplementary Figures.

## Data Availability

Images showing gels/blots and fluorescence images are compliance with the digital image and integrity policies. Original gels/blots used in figures are included in the Supplementary Information. The datasets generated and/or analysed during the current study are available from the corresponding author on reasonable request.
