# Author Correction: Amyloid-β impairs mitochondrial dynamics and autophagy in Alzheimer’s disease experimental models

**DOI:** 10.1038/s41598-023-46106-y

**Published:** 2023-11-07

**Authors:** Macarena de la Cueva, Desiree Antequera, Lara Ordoñez‑Gutierrez, Francisco Wandosell, Antonio Camins, Eva Carro, Fernando Bartolome

**Affiliations:** 1 Network Center for Biomedical Research in Neurodegenerative Diseases (CIBERNED), Madrid, Spain; 2 Group of Neurodegenerative Diseases, Hospital Universitario 12 de Octubre Research Institute (imas12), 28041 Madrid, Spain; 3 Centro de Biología Molecular “Severo Ochoa” (CSIC-UAM), Universidad Autónoma de Madrid, 28049 Madrid, Spain; 4 Department of Pharmacology, Toxicology and Therapeutic Chemistry, Faculty of Pharmacy & Food Sciences, University of Barcelona, Barcelona, Spain; 5 Institut de Neurociències (UBNeuro), University of Barcelona, Barcelona, Spain

Correction to: *Scientifc Reports*, 10.1038/s41598-022-13683-3, published online 16 June 2022

The Funding section in the original version of this Article was incomplete.

“This study was supported by Grants from Instituto de Salud Carlos III (PI18/00118; PI21/00183; CP20/00007), FEDER, Comunidad de Madrid (S2017/BMD-3700; NEUROMETAB-CM), and CIBERNED (CB07/502). In addition FW was supported by grants from ISCIII-CIBERNED (CB06/05/0067) and I+D+i-RETOS- RTI2018-096303-B.”

now reads:

“This study has been funded by Instituto de Salud Carlos III (ISCIII) through the projects “PI18/00118”, “PI21/00183” and “CP20/00007” and co-funded by the European Union, Comunidad de Madrid (S2017/BMD-3700; NEUROMETAB-CM), and CIBERNED (CB07/502). In addition FW was supported by grants from ISCIII-CIBERNED (CB06/05/0067) and I+D+i-RETOS- RTI2018-096303-B.”

The original Article has been corrected.

