# Supplementary material for: Amyloid-β impairs mitochondrial dynamics and autophagy in Alzheimer’s disease experimental models
Source: Sci Rep. 2022 Jun 16;12:10092. doi: 10.1038/s41598-022-13683-3 (PMC9203760; doi:10.1038/s41598-022-13683-3)
Supplement: Supplementary file 1 — Supplementary Figures. [file 41598_2022_13683_MOESM1_ESM.pdf]

Supplementary Information

Amyloid-β impairs mitochondrial dynamics and autophagy in Alzheimer's disease experimental models.

Macarena de la Cueva, Desiree Antequera, Lara Ordoñez-Gutierrez, Francisco Wandosell, Antonio Camins, Eva Carro, Fernando Bartolome.

SUPLEMENTARY MATERIAL

Supplementary material includes Supplementary Figures 1-21.

LEGENDS OF SUPPLEMENTARY FIGURES

Supplementary Figure 1 – Aβ40 and Aβ42 levels in cerebral cortex and hippocampus from APP/PS1 mice. Human Aβ40 and Aβ42 levels were estimated in the brain tissue from 3-, 6- and 12-month-old APP/PS1 mice using ELISA.

a) Aβ40 levels in cerebral cortex.

b) Aβ40 levels in hippocampus.

c) Aβ42 levels in cerebral cortex.

d) Aβ42 levels in hippocampus.

In all cases Aβ peptide levels are expressed as percentage of the mean ± SEM and they are referred to the 3-month-old mice group. *p<0.05, **p<0.01, ***p<0.001, ****p<0.0001. Statistical significance was assessed by one-way ANOVA followed by Fischer's post hoc test for multiple comparisons.

Supplementary Figure 2 – TFAM levels in SH-SY5Y cells and primary neurons. Histograms (top panels) showing the TFAM levels in SH-SY5Y cells (left panels) and rat primary neurons (right panels) treated with 1 µM oligomerised Aβ42 for 24 h. Representative images of immunoblots are presented at the bottom panels. Data are expressed as mean ± SEM; SH-SY5Y cells: n = 3, primary neurons: n = 3. *$p<0.05$. Statistical significance was assessed by student's t-test.

Supplementary Figure 3 – Full-length blots from Figure 1A-B.

a) Full-length blots from Figure 1A corresponding to CxVβ and β-actin as loading control. In both, dashed lines show the cropped sections shown in Figure 1A.

b) Full-length blots from Figure 1B corresponding to CxVβ and β-actin as loading control. In both, dashed lines show the cropped sections shown in Figure 1B.

Supplementary Figure 4 – Full-length blots of additional exposures for β-actin in Figure 1B.

Supplementary Figure 5 – Full-length blots from Figure 1C.

a) Full-length blots from Figure 1C corresponding to CxVβ. Dashed lines show the cropped section shown in Figure 1C.

b) Full-length blots of additional exposure for CxVβ in Figure 1C.

c) Full-length blots from Figure 1C corresponding to β-actin as loading control. Dashed lines show the cropped section shown in Figure 1C.

Supplementary Figure 6 – Full-length blots from Figure 1D.

Full-length blots of selected exposure from Figure 1D corresponding to CxVβ and β-actin as loading control. In both, dashed lines show the cropped sections shown in Figure 1D. Blot membrane was cut at 37 kDa marker prior to antibody hybridisation. For CxVβ, during digital exposure the blot membrane was covered at 75 kDa to avoid the signal of previous antibody incubations.

Supplementary Figure 7 – Full-length blots of additional exposures for CxVβ in Figure 1D. Blot membrane was cut at 37 kDa marker prior to antibody hybridisation. For CxVβ, during digital exposure the blot membrane was covered at 75 kDa to avoid the signal of previous antibody incubations.

Supplementary Figure 8 – Full-length blots of additional exposures for β-actin in Figure 1D. Blot membrane was cut at 37 kDa marker prior to antibody hybridisation.

Supplementary Figure 9 – Full-length blots from Figure 2A-B.

a) Full-length blots from Figure 2A corresponding to PGC-1α and β-actin as loading control of APP/PS1 cerebral cortex. In both, dashed lines show the cropped sections shown in Figure 2A.

b) Full-length blots from Figure 2B corresponding to PGC-1α and β-actin as loading control of APP/PS1 hippocampus. In both, dashed lines show the cropped sections shown in Figure 2B.

Supplementary Figure 10 – Full-length blots from Figure 2C-D.

a) Full-length blots from Figure 2C corresponding to PGC-1α and β-actin as loading control in rat primary neurons. In both, dashed lines show the cropped sections shown in Figure 2C.

b) Full-length blots from Figure 2D corresponding to PGC-1α and β-actin as loading control in SH-SY5Y cells. In both, dashed lines show the cropped sections shown in Figure 2D.

Supplementary Figure 11 – Full-length blots from Figure 3A.

a) Full-length blots from Figure 3A (left panel) corresponding to Mfn1 and β-actin as loading control of APP/PS1 cerebral cortex. In both, dashed lines show the cropped sections shown in Figure 3A (left panel).

b) Full-length blots from Figure 3A (right panel) corresponding to Mfn1 and β-actin as loading control of APP/PS1 hippocampus. In both, dashed lines show the cropped sections shown in Figure 3A (right panel).

Supplementary Figure 12 – Full-length blots from Figure 3B.

a) Full-length blots from Figure 3B (left panel) corresponding to Mfn2 and β-actin as loading control of APP/PS1 cerebral cortex. In both, dashed lines show the cropped sections shown in Figure 3B (left panel). During digital exposure the blot membrane was covered below 75 kDa to avoid the signal of previous antibody incubations.

b) Full-length blots from Figure 3B (right panel) corresponding to Mfn2 and β-actin as loading control of APP/PS1 hippocampus. In both, dashed lines show the cropped sections shown in Figure 3B (right panel).

Supplementary Figure 13 – Full-length blots from Figure 3C.

a) Full-length blots from Figure 3C (left panel) corresponding to Opa1 and β-actin as loading control of APP/PS1 cerebral cortex. In both, dashed lines show the cropped sections shown in Figure 3C (left panel). To avoid the signal of previous antibody incubations, the blot membrane was covered below 75 kDa during digital exposure for Opa1 and above 50 kDa for the digital exposure for β-actin.

b) Full-length blots from Figure 3C (right panel) corresponding to Opa1 and β-actin as loading control of APP/PS1 hippocampus. In both, dashed lines show the cropped sections shown in Figure 3C (right panel).

Supplementary Figure 14 – Full-length blots from Figure 3D.

a) Full-length blots from Figure 3D (left panel) corresponding to Drp1 and β-actin as loading control of APP/PS1 cerebral cortex. In both, dashed lines show the cropped sections shown in Figure 3D (left panel).

b) Full-length blots from Figure 3D (right panel) corresponding to Drp1 and β-actin as loading control of APP/PS1 hippocampus. In both, dashed lines show the cropped sections shown in Figure 3D (right panel). Blot membrane was cut at 37 kDa marker prior to antibody hybridisation. During digital exposure of Drp1 the blot membrane was covered below 75 kDa to avoid the signal of previous antibody incubations.

c) Full-length blots of additional exposures for Drp1 in hippocampus of APP/PS1 mice from Figure 3D (right panel). During digital exposure of Drp1 the blot membrane was covered below 75 kDa to avoid the signal of previous antibody incubations.

Supplementary Figure 15 – Full-length blots from Figure 3E (left panel).

Full-length blots of selected exposure from Figure 3E (left panel) corresponding to Mfn1, Mfn2, Opa1, Drp1 and β-actin as loading control in rat primary neurons. In all cases, dashed

lines show the cropped sections shown in Figure 3E (left panel). During digital exposure of Drp1 the blot membrane was covered below 75 kDa to avoid the signal of previous antibody incubations.

Supplementary Figure 16 – Full-length blots from Figure 3E (right panel).

Full-length blots of selected exposure from Figure 3E (right panel) corresponding to Mfn1, Mfn2, Opa1, Drp1 and β-actin as loading control in SH-SY5Y cells. In all cases, dashed lines show the cropped sections shown in Figure 3E (right panel).

Supplementary Figure 17 – Full-length blots of additional exposures from Figure 3E (right panel).

Full-length blots of additional exposure from Figure 3E (right panel) corresponding to Mfn1, Opa1, Drp1 and β-actin as loading control in SH-SY5Y cells. In all cases, dashed lines show the cropped sections shown in Figure 3E (right panel).

Supplementary Figure 18 – Full-length blots from Figure 5E.

a) Full-length blots from Figure 5E corresponding to p62 (top left) and β-actin (bottom left) as loading control and LC3-I/II and β-actin (right panel) in neuronal primary cell cultures. In all cases, dashed lines show the cropped sections shown in Figure 5E. During digital exposure for β-actin the blot membrane was covered above 50 kDa to avoid the signal of previous antibody incubations.

b) Full-length blots of additional exposures from Figure 5E (left panel) corresponding to β-actin. In both, dashed lines show the cropped sections shown in Figure 5E (left panel). During digital exposure the blot membrane was covered above 50 kDa to avoid the signal of previous antibody incubations.

Supplementary Figure 19 – Full-length blots from Figure 5F.

Full-length blots from Figure 5F corresponding to p62 and LC3 I/II and β-actin as loading control in SH-SY5Y cells. In all cases, dashed lines show the cropped sections shown in Figure 5F. During digital exposure for LC3 I/II the blot membrane was covered above 37 kDa to avoid the signal of previous antibody incubations.

Supplementary Figure 20 – Full-length blots from Figure 5G.

a) Full-length blots from Figure 5G (first two panels from left to right) corresponding to p62 and β-actin as loading control in cerebral cortex from APP/PS1 mice. In both, dashed lines show the cropped sections shown in Figure 5G (first two panels from left to right).

b) Full-length blots from Figure 5G (second two panels from left to right) corresponding to p62 and β-actin as loading control in hippocampus from APP/PS1 mice. In both, dashed lines show the cropped sections shown in Figure 5G (second two panels from left to right).

Supplementary Figure 21 – Full-length blots from Figure 5G.

a) Full-length blots from Figure 5G (third two panels from left to right) corresponding to LC3 I/II and β-actin as loading control in cerebral cortex from APP/PS1 mice. In both, dashed lines show the cropped sections shown in Figure 5G (third two panels from left to right). During digital exposure for LC3 I/II the blot membrane was covered above 25 kDa to avoid the signal of previous antibody incubations.

b) Full-length blots from Figure 5G (fourth two panels from left to right) corresponding to LC3 I/II and β-actin as loading control in hippocampus from APP/PS1 mice. In both, dashed lines show the cropped sections shown in Figure 5G (fourth two panels from left to right). During digital exposure for LC3 I/II the blot membrane was covered above 25 kDa to avoid the signal of previous antibody incubations.

Supplementary Figure 22 – Full-length blots from supplementary Figure 2.

a) Full-length blots from Supplementary Figure 2 corresponding to TFAM and β-actin as loading control in SH-SY5Y cells (left panel). In both cases, dashed lines show the cropped sections shown in the Supplementary Figure 2, left panel.

b) Full-length blots from Supplementary Figure 2 corresponding to TFAM and β-actin as loading control in primary neurons (right panel). In both cases, dashed lines show the cropped sections shown in the Supplementary Figure 2, right panel.

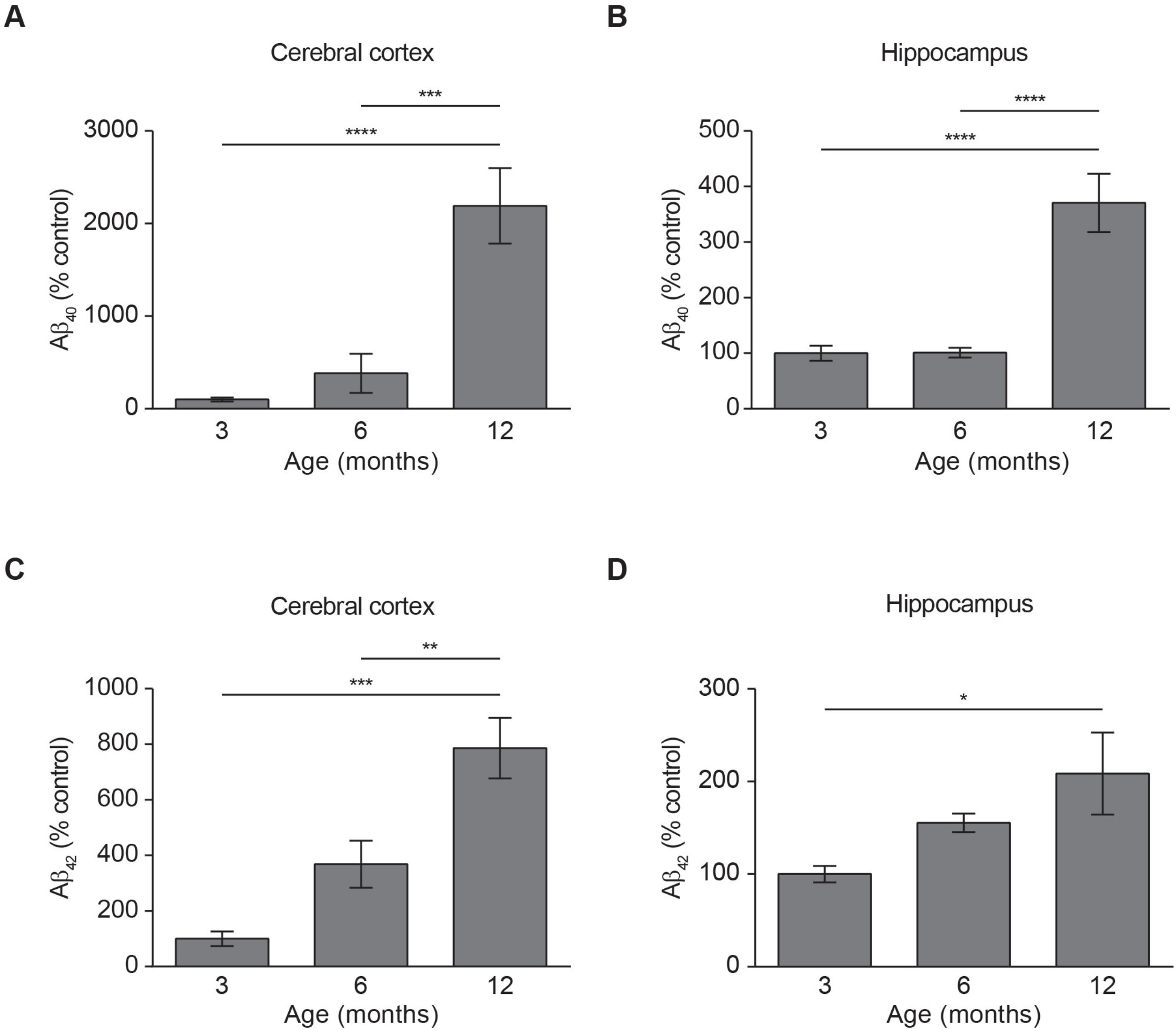

Supplementary Figure 1

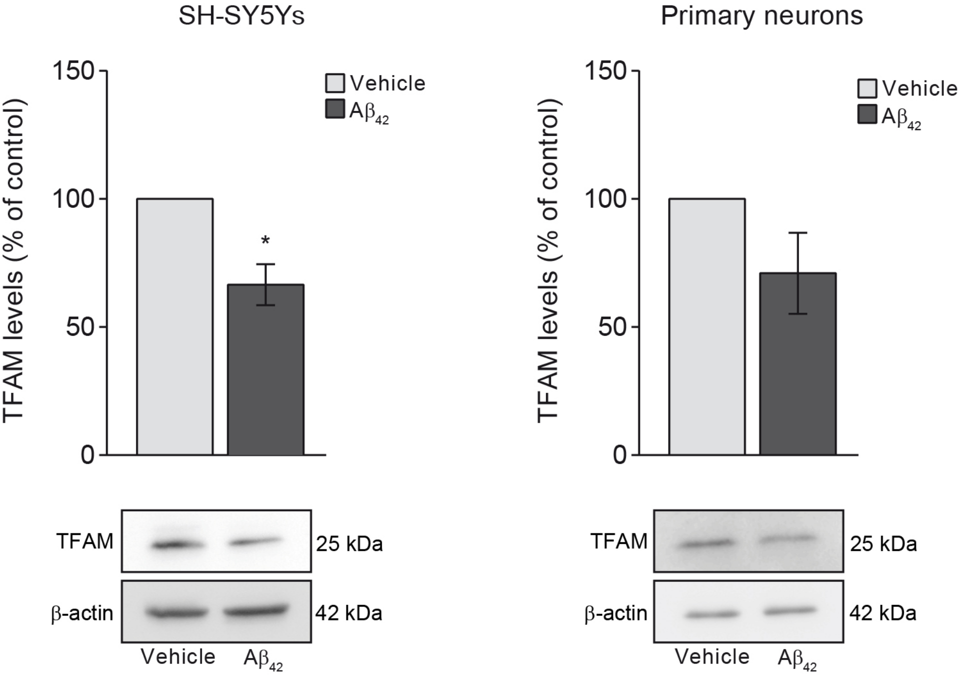

Supplementary Figure 2

a

Figure 1A

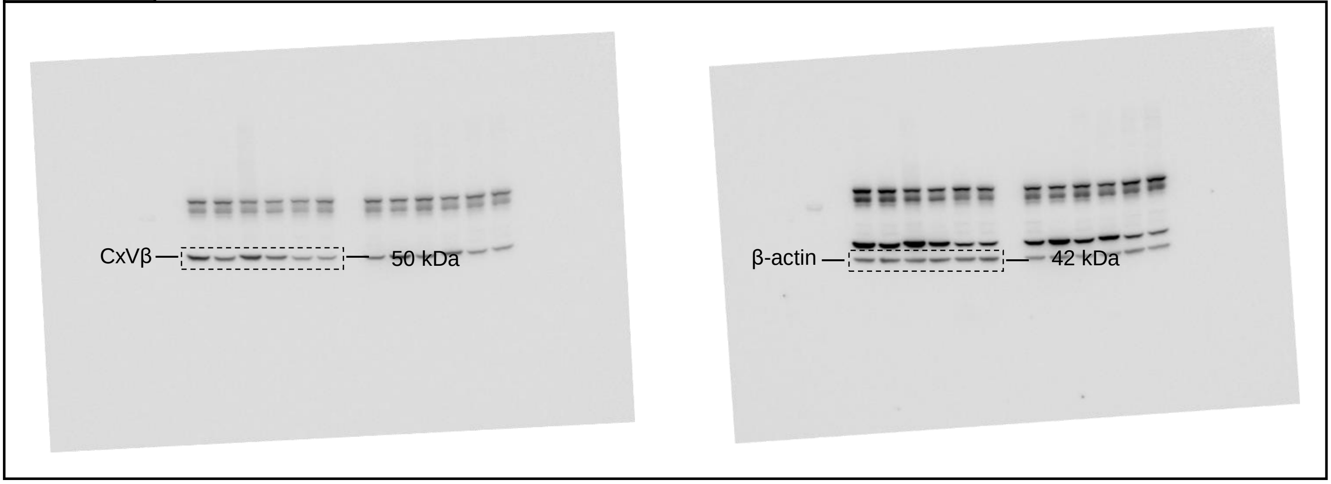

b

Figure 1B (including selected exposure for β-actin)

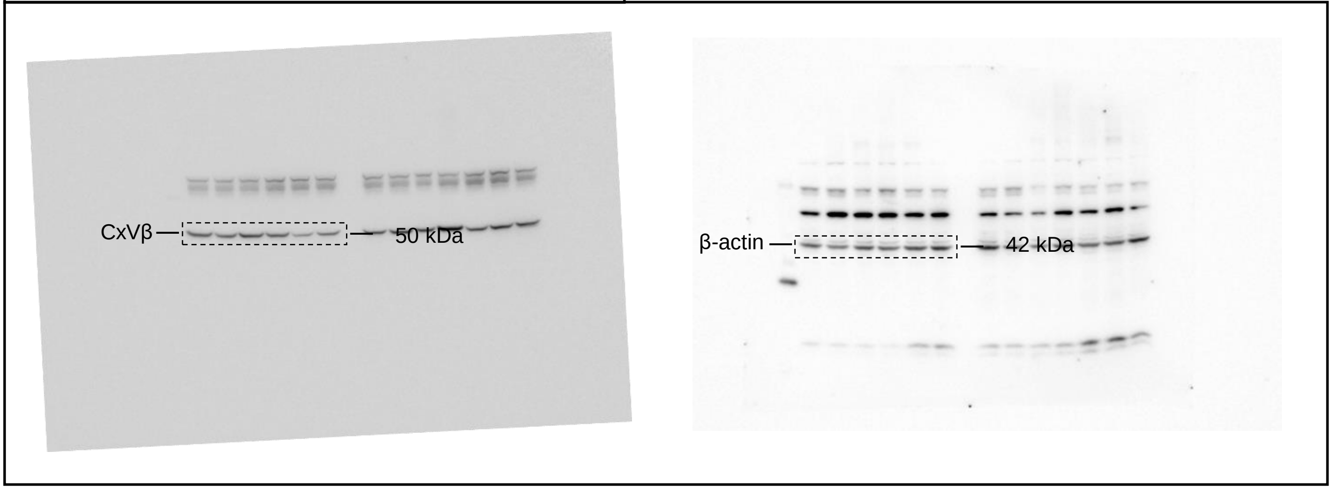

Supplementary Figure 3

Figure 1B (additional exposures for β-actin)

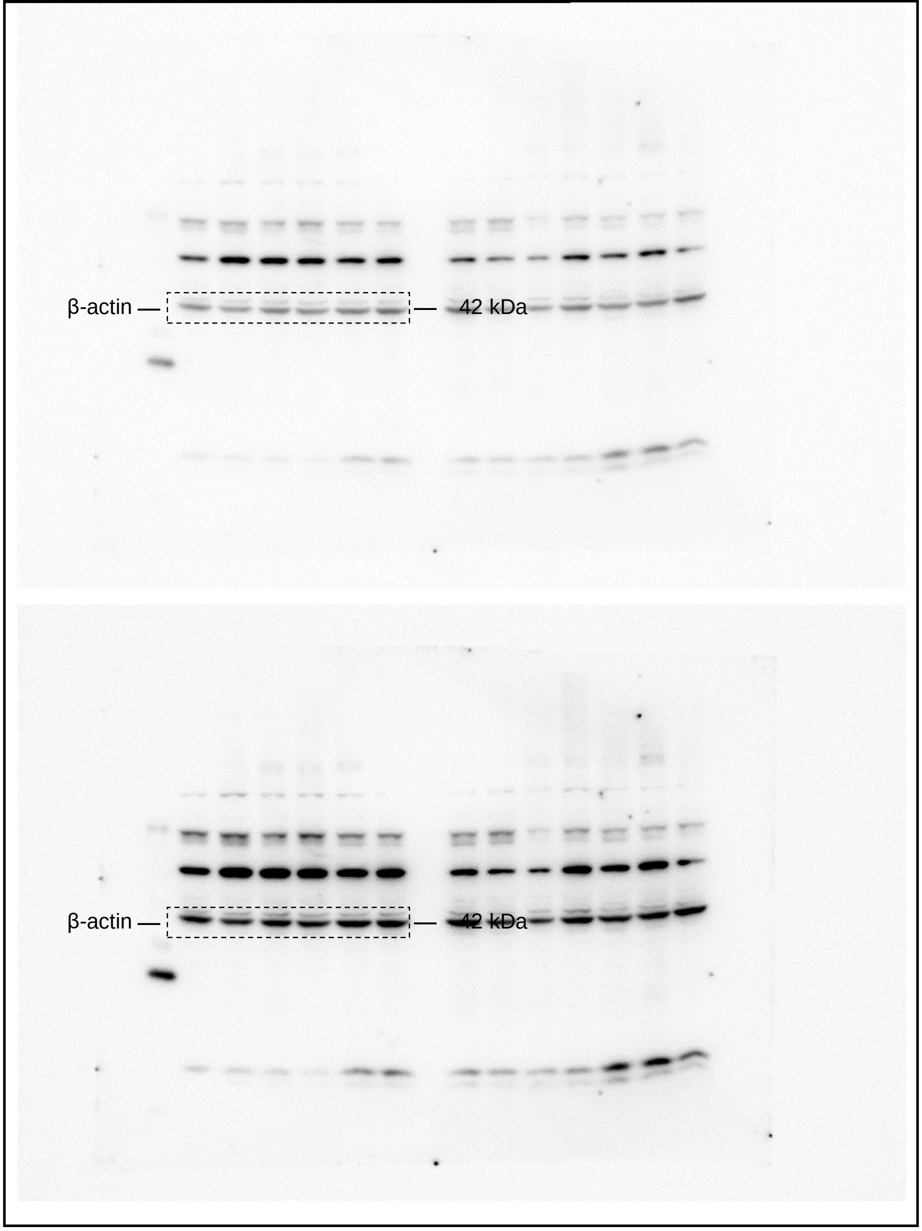

Supplementary Figure 4

a

Figure 1C (selected exposure for CxVβ)

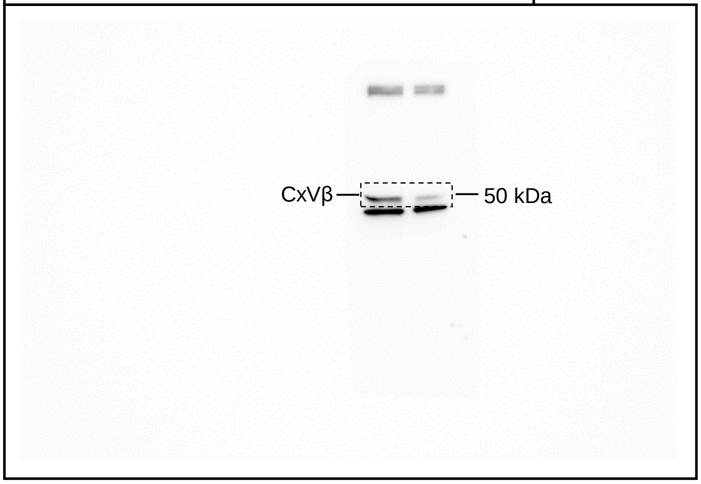

b

Figure 1C (additional exposure for CxVβ)

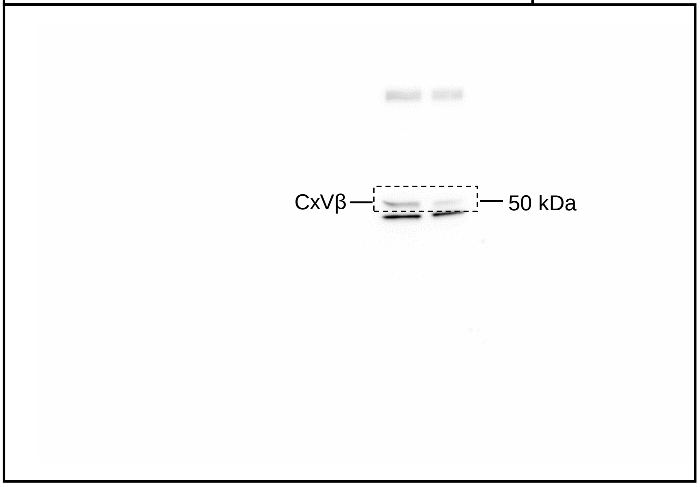

c

Figure 1C (selected exposure for β-actin)

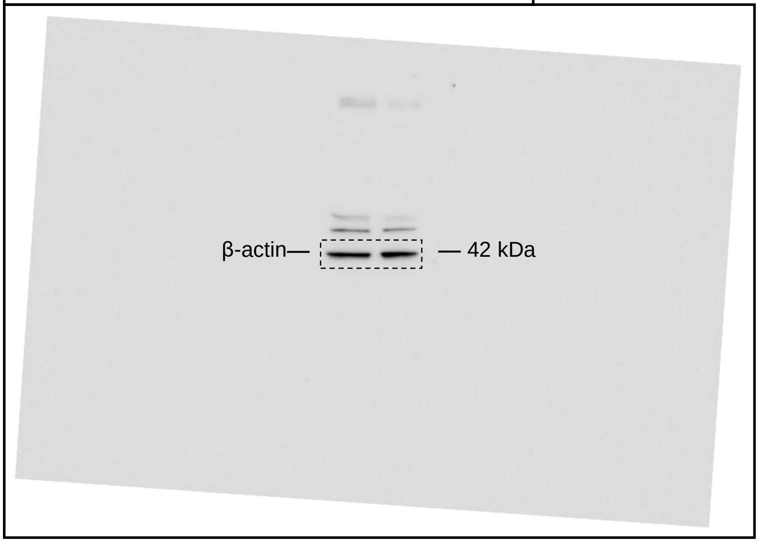

Supplementary Figure 5

Figure 1D (selected exposure for CxVβ and β-actin

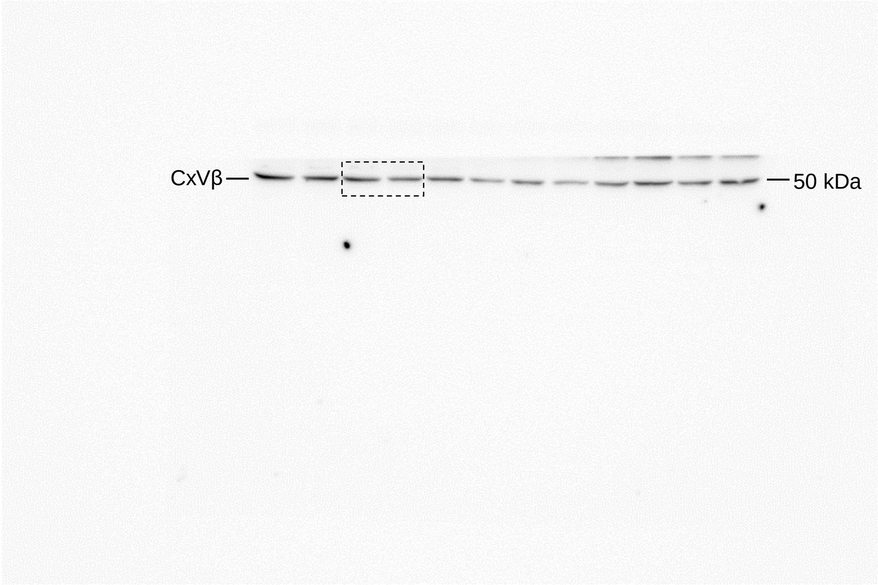

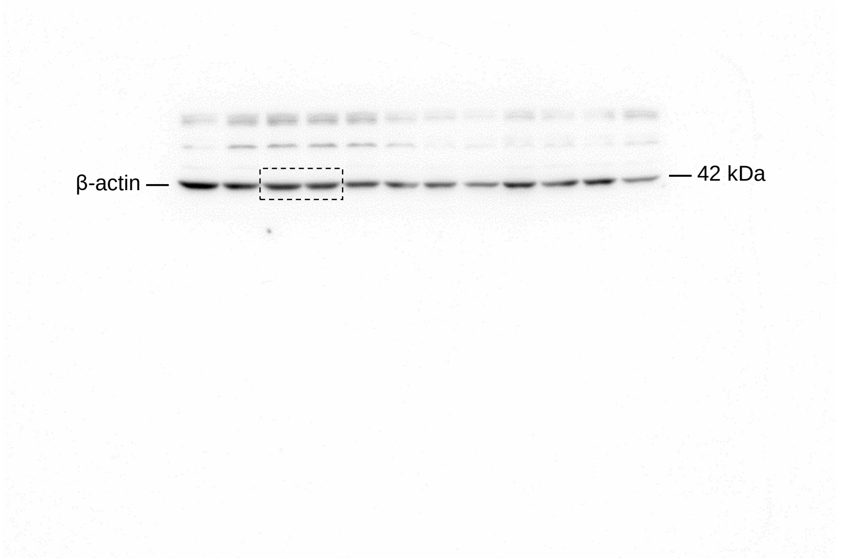

Supplementary Figure 6

Figure 1D (additional exposures for CxVβ

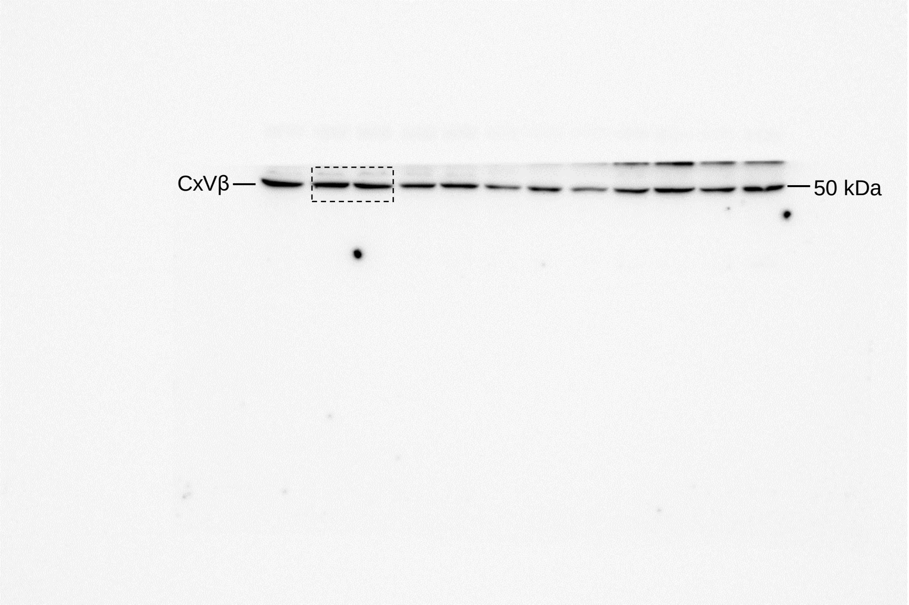

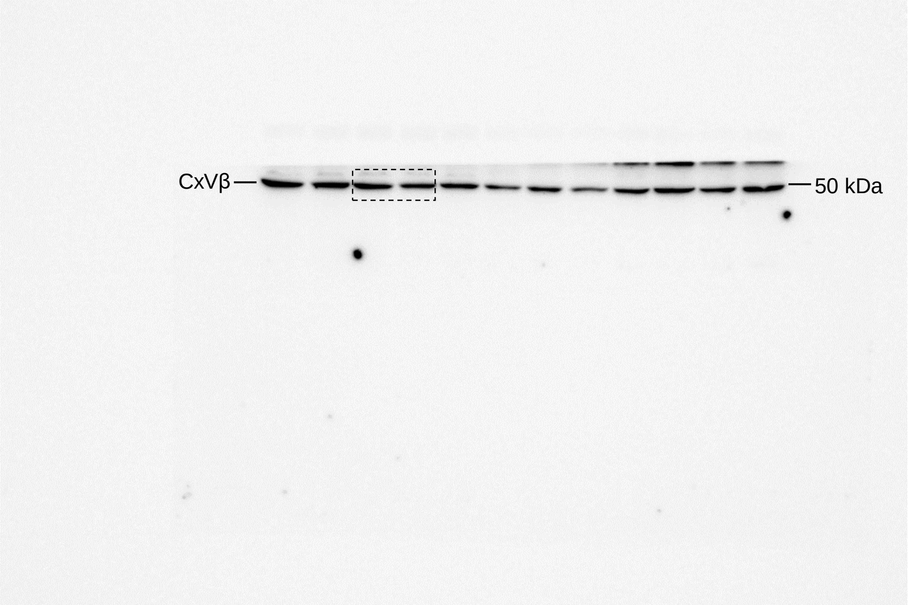

Supplementary Figure 7

Figure 1D (additional exposures for β-actin)

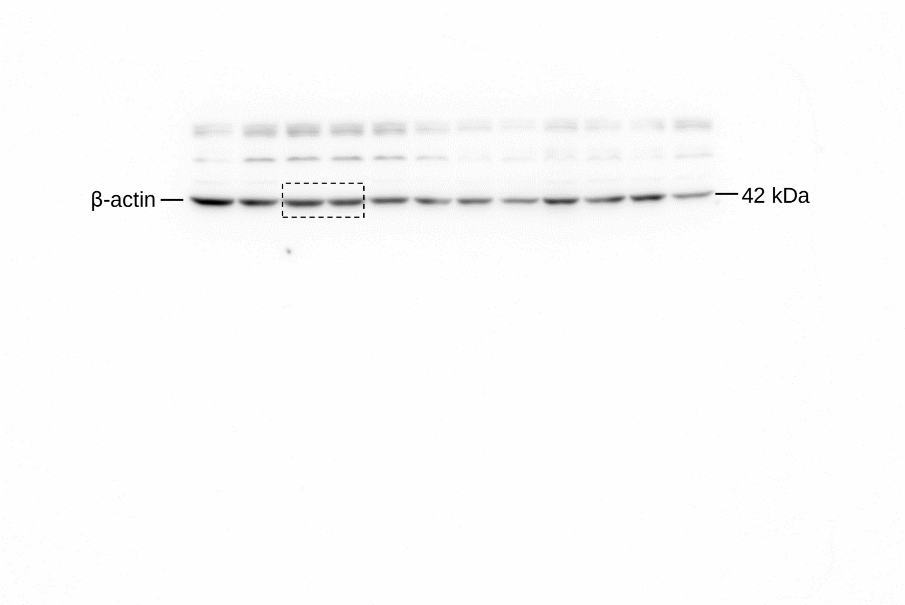

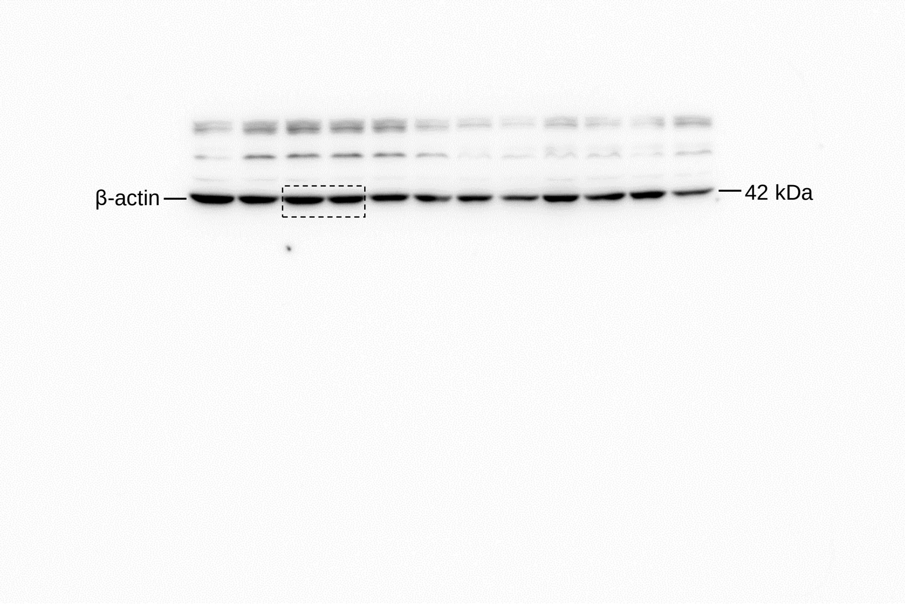

Supplementary Figure 8

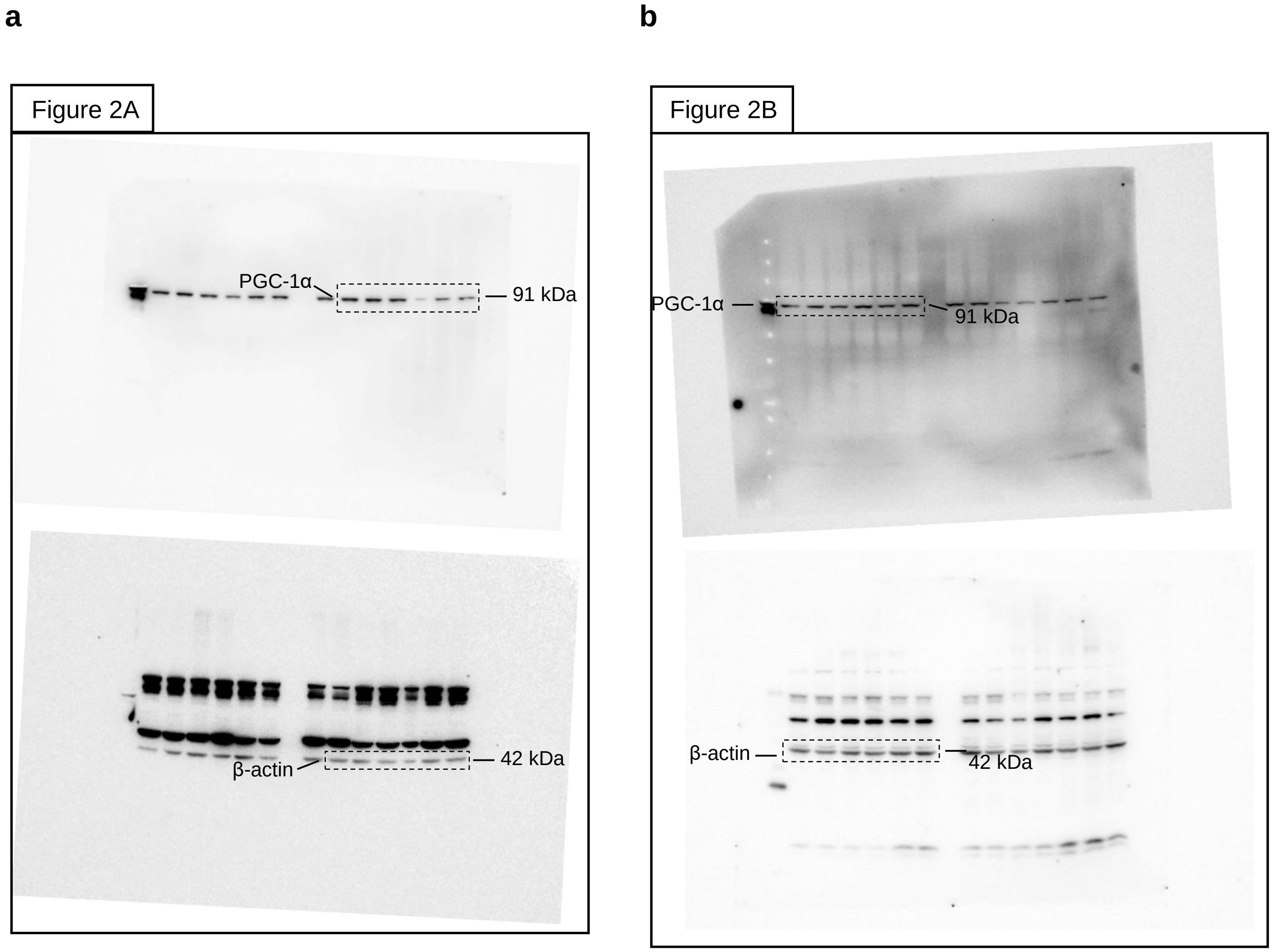

Supplementary Figure 9

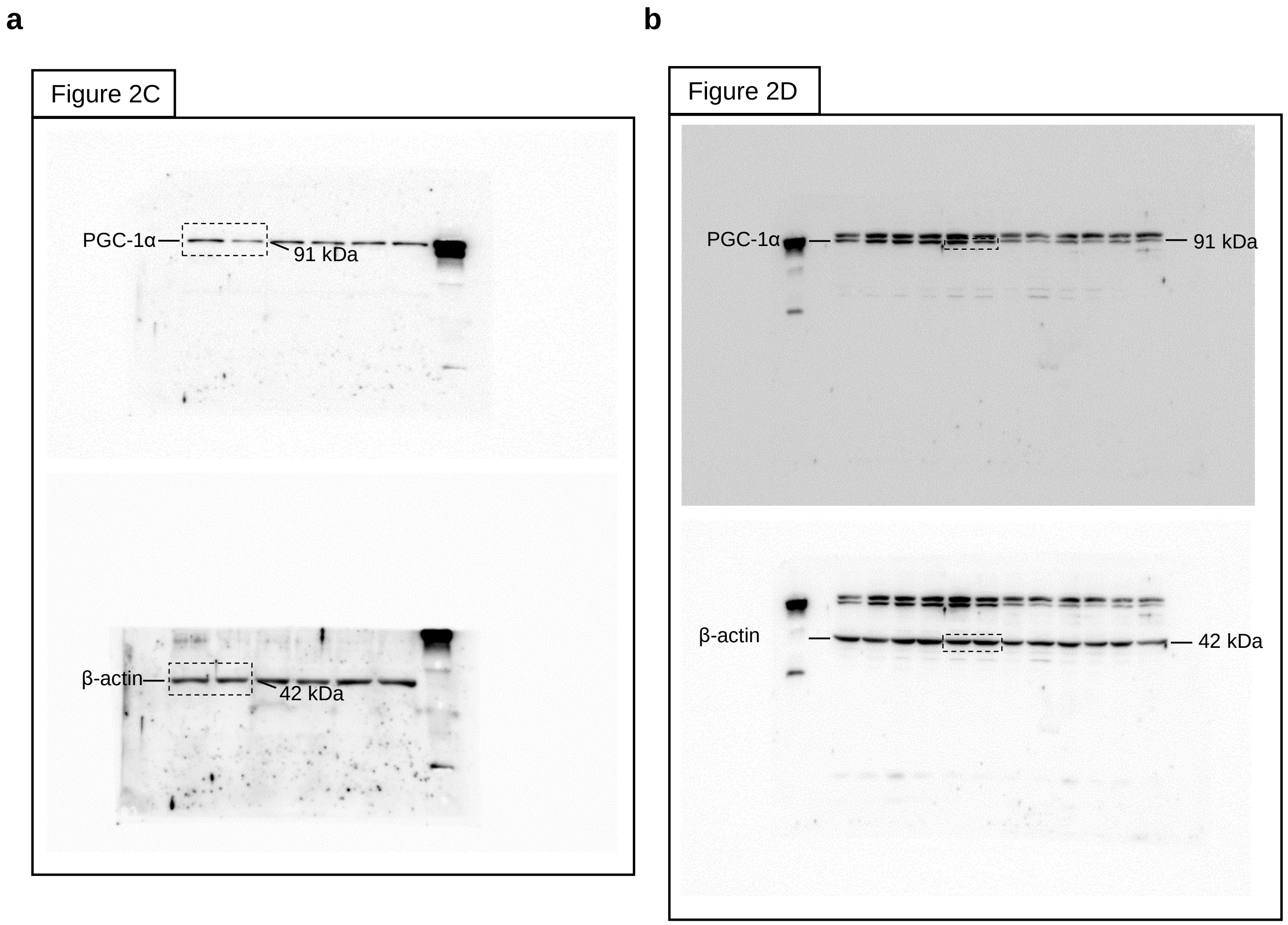

Supplementary Figure 10

a

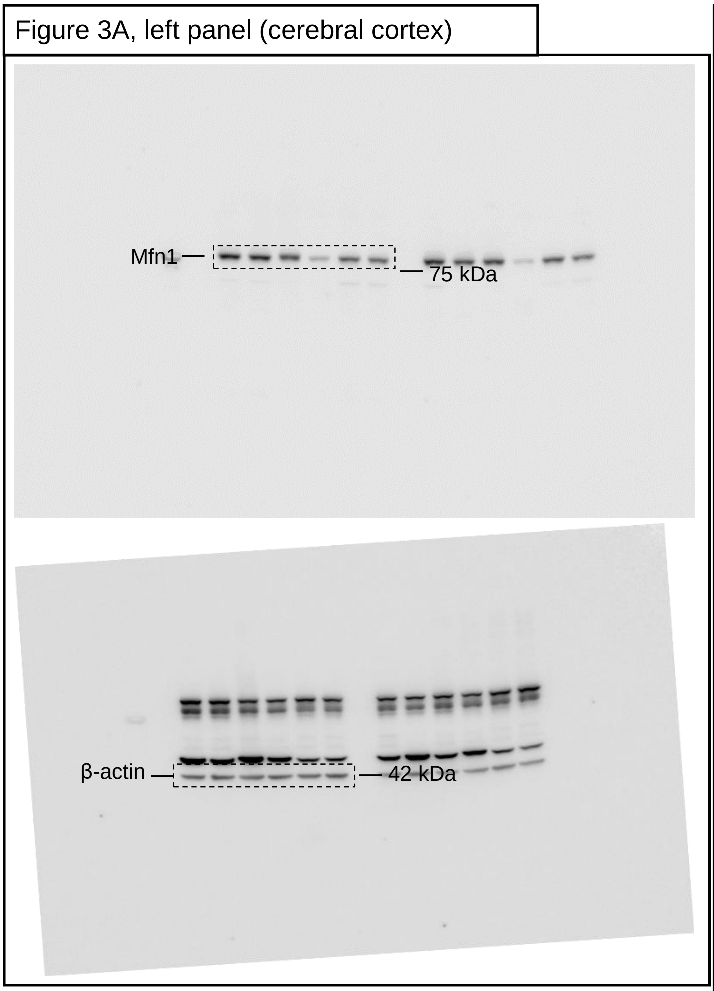

b

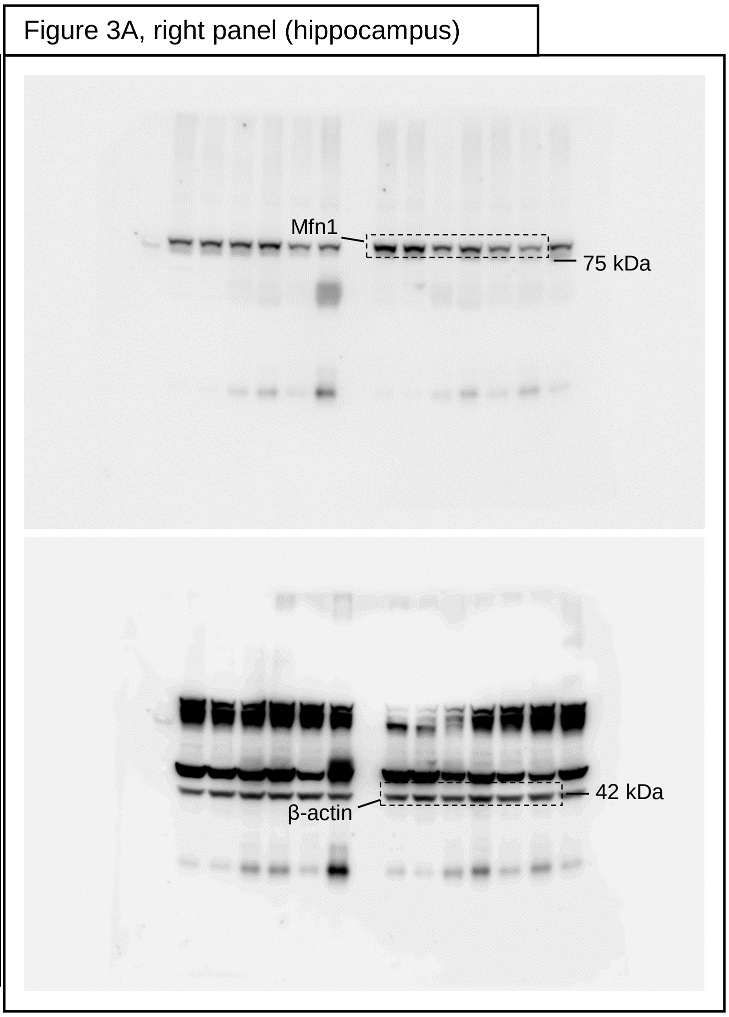

Supplementary Figure 11

a

Figure 3B, left panel (cerebral cortex)

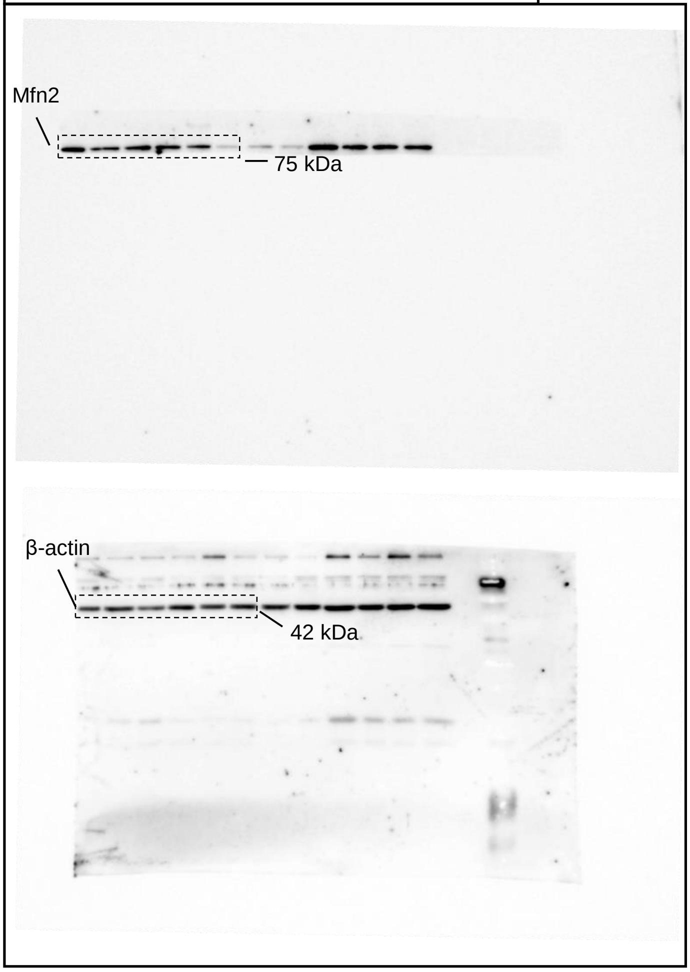

b

Figure 3B, right panel (hippocampus)

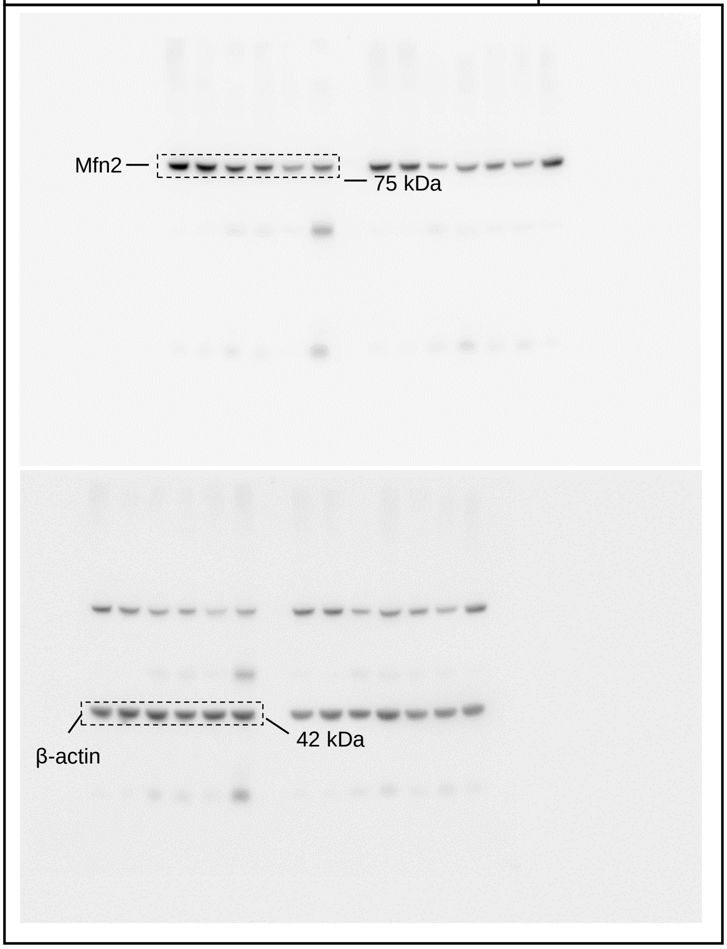

Supplementary Figure 12

a

Figure 3C, left panel (cerebral cortex)

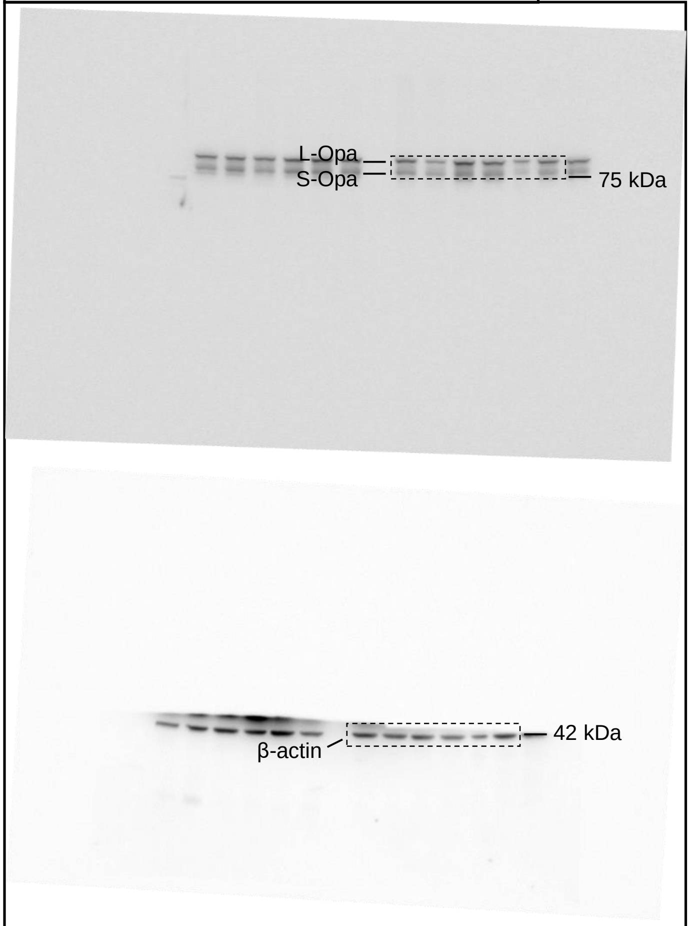

b

Figure 3C, right panel (hippocampus)

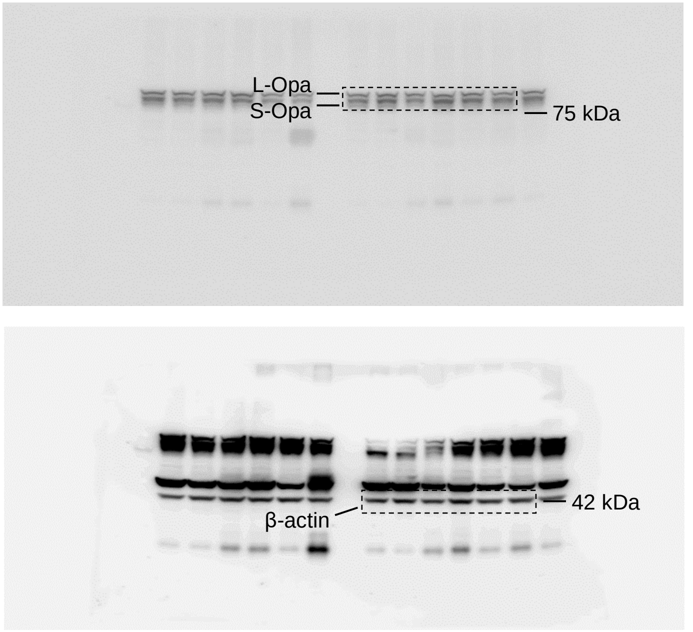

Supplementary Figure 13

a

Figure 3D, left panel (cerebral cortex)

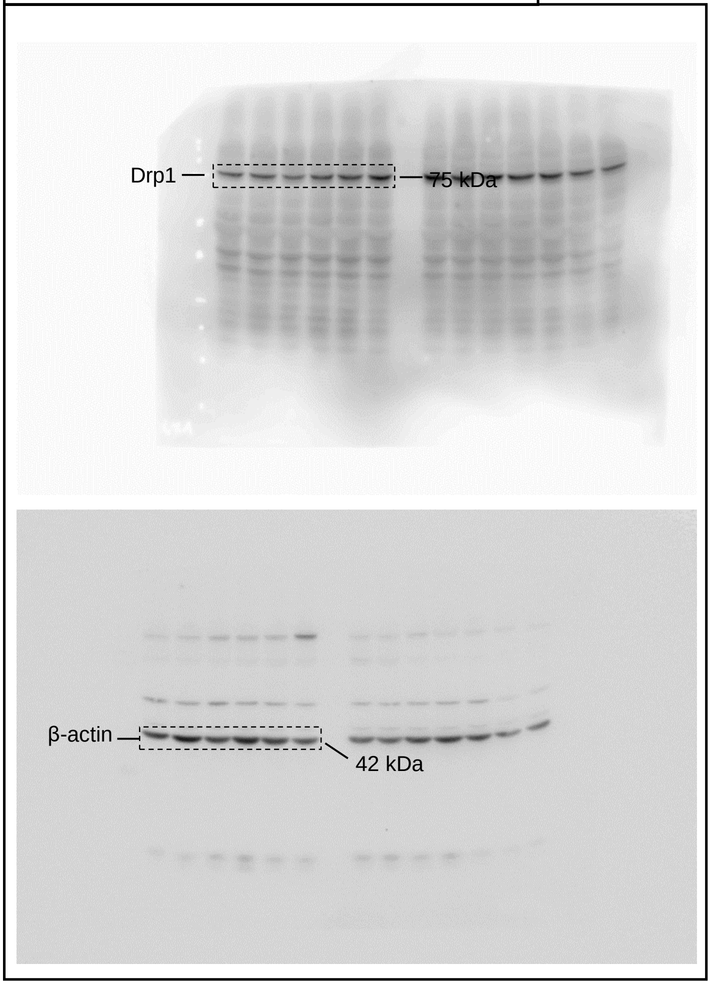

b

Figure 3D, right panel (hippocampus)

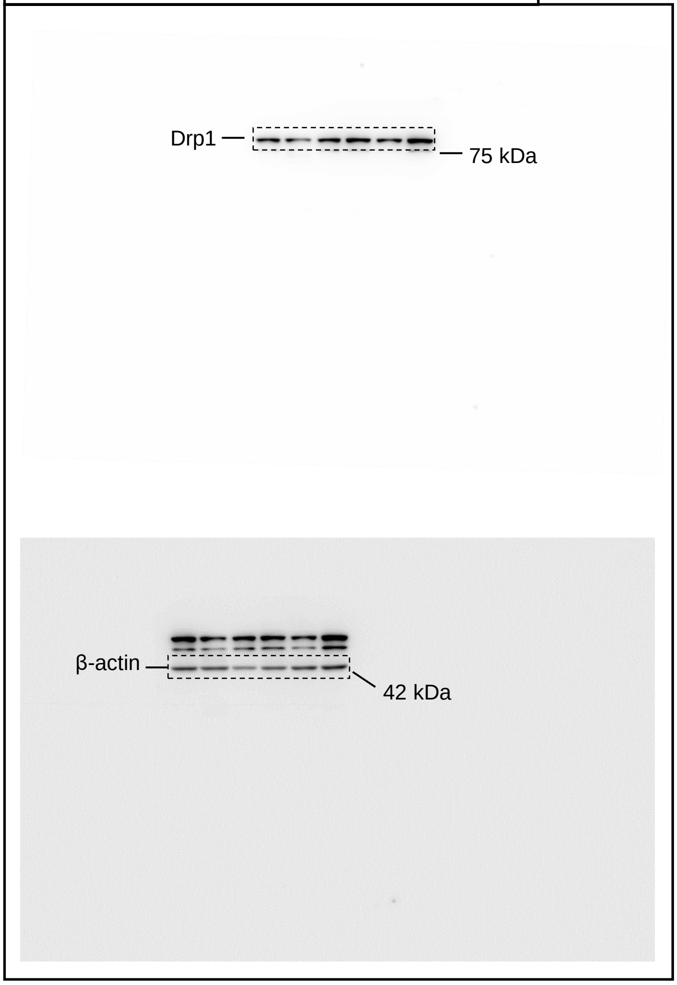

c

Figure 3D, right panel (hippocampus): additional axposures of Drp1

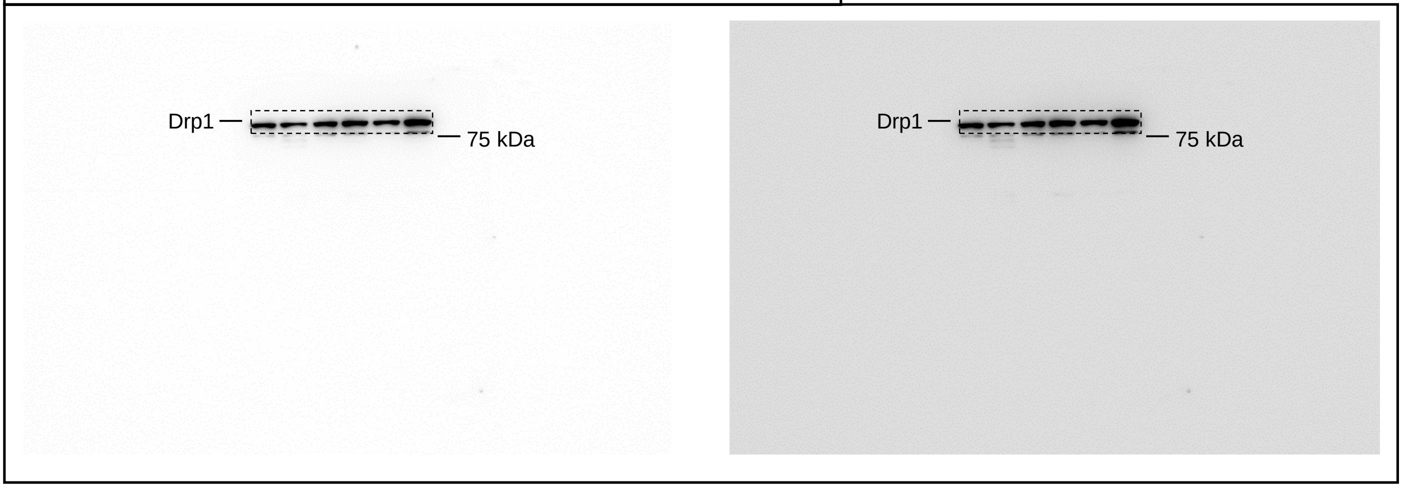

Supplementary Figure 14

Figure 3E, primary neurons

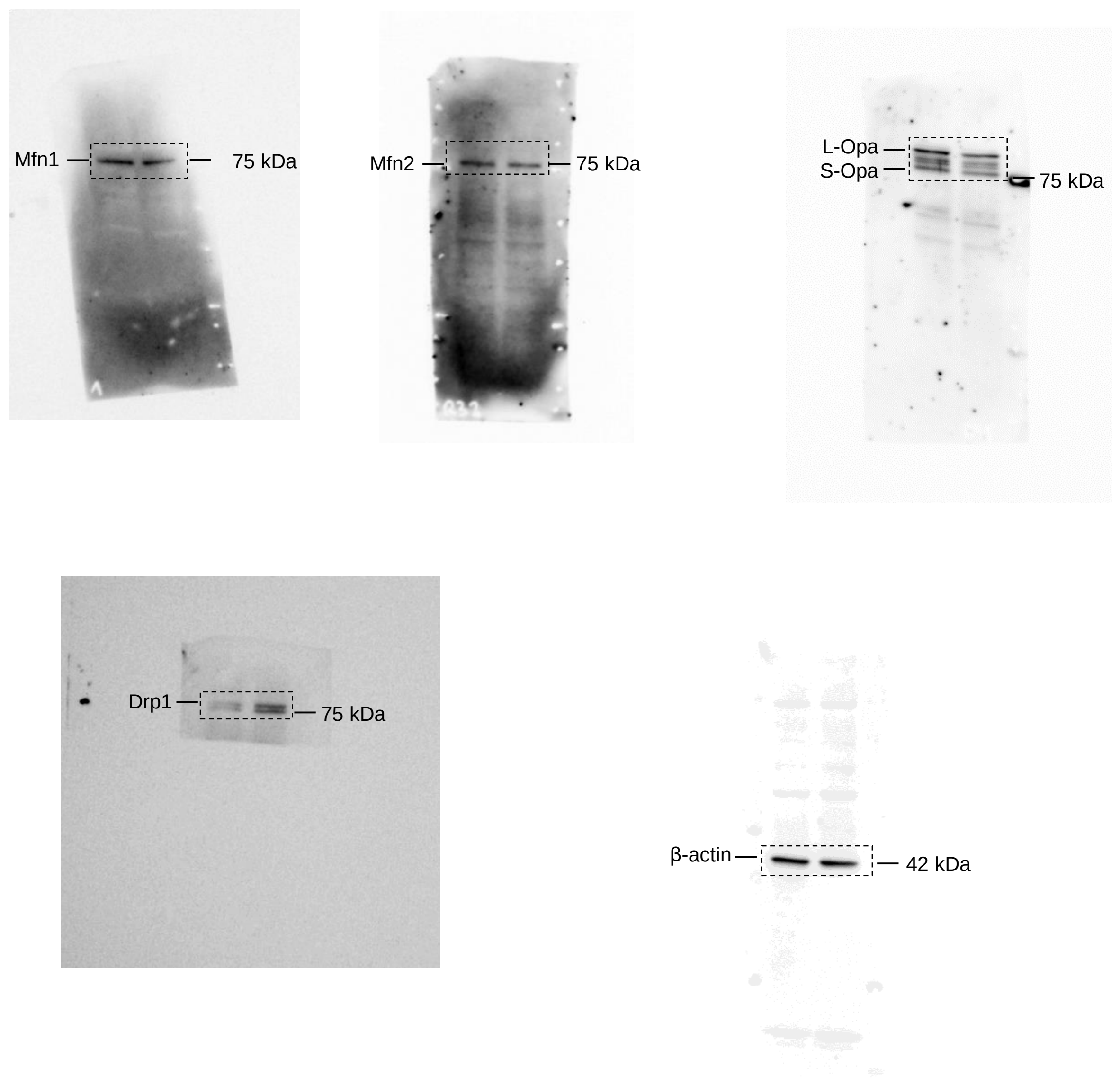

Supplementary Figure 15

Figure 3E, SH-SY5Y cells (selected exposures)

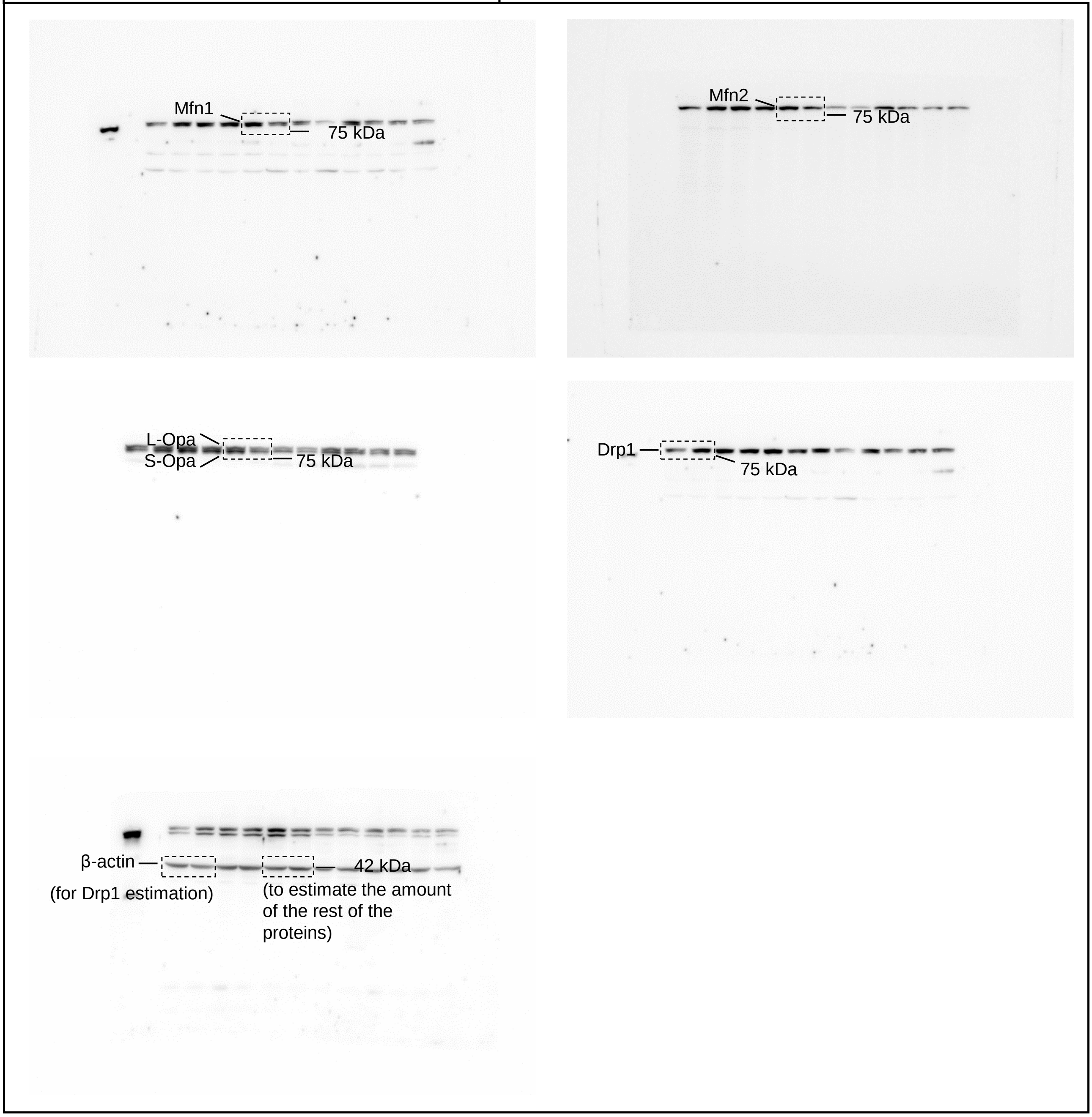

Supplementary Figure 16

Figure 3E, SH-SY5Y cells (additional exposures for Mfn1, Opa 1 Drp1 and β-actin)

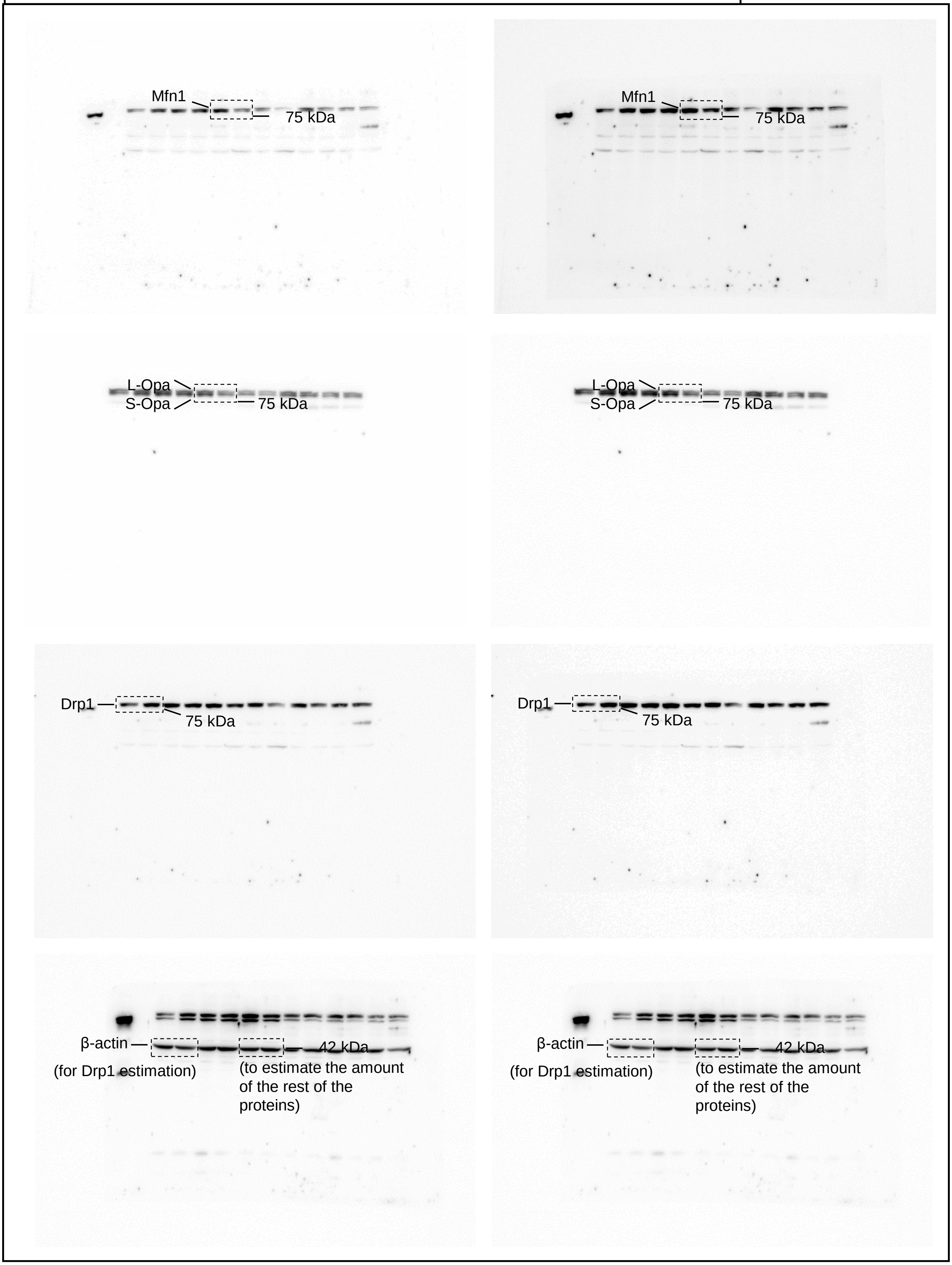

Supplementary Figure 17

a

Figure 5E, p62 and LC3 I/II in primary neurons and selected exposure for β-actin

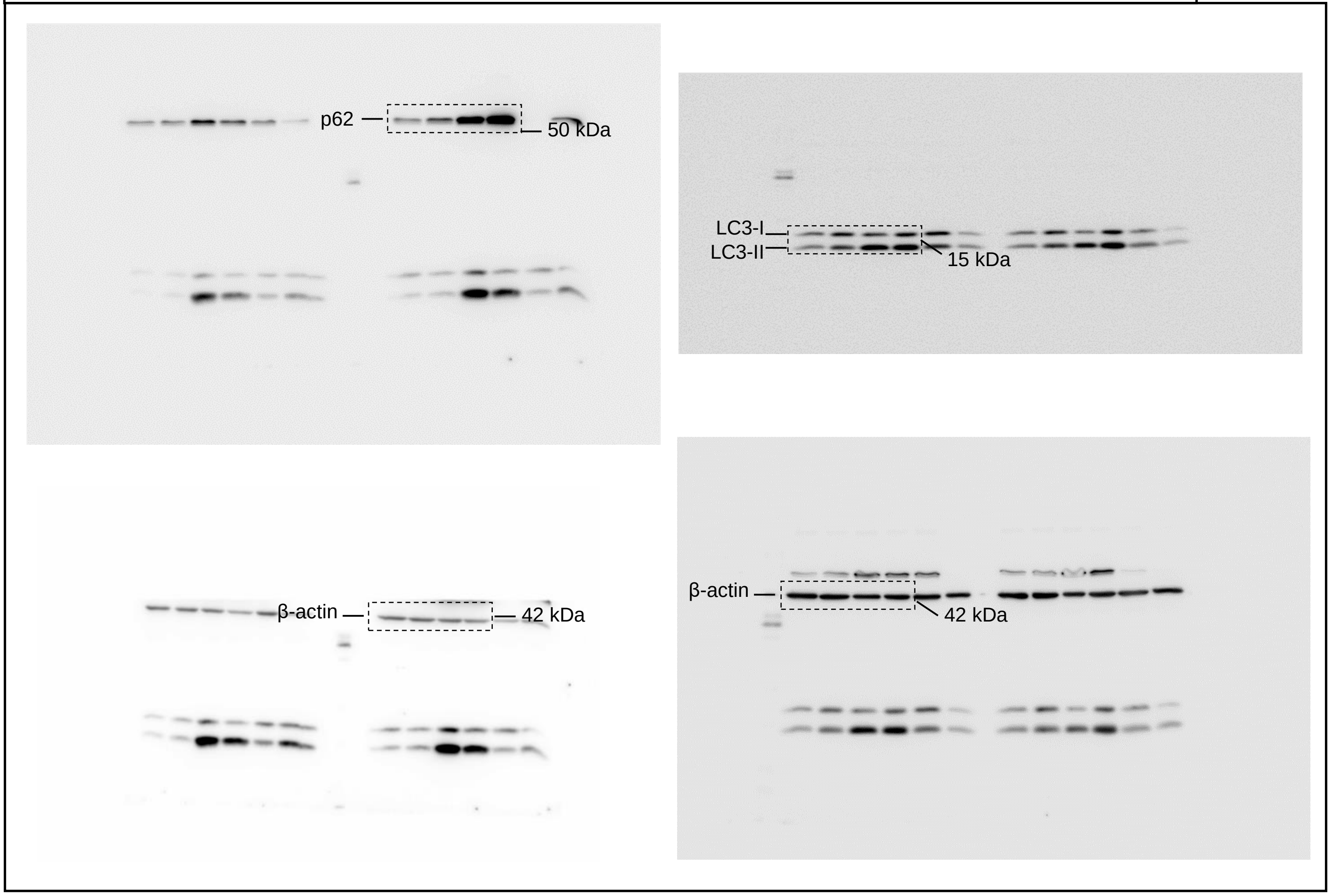

b

Figure 5E (left panel), primary neurons (additional exposures for β-actin)

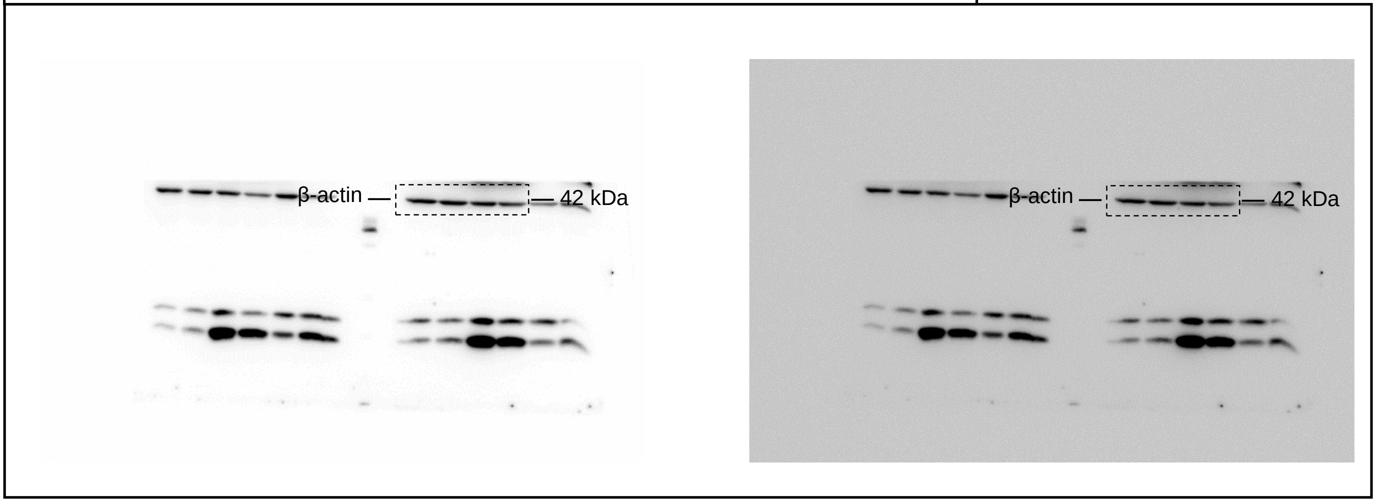

Supplementary Figure 18

Figure 5F, p62 and LC3 I/II in SH-SY5Y cells and selected exposure for β-actin

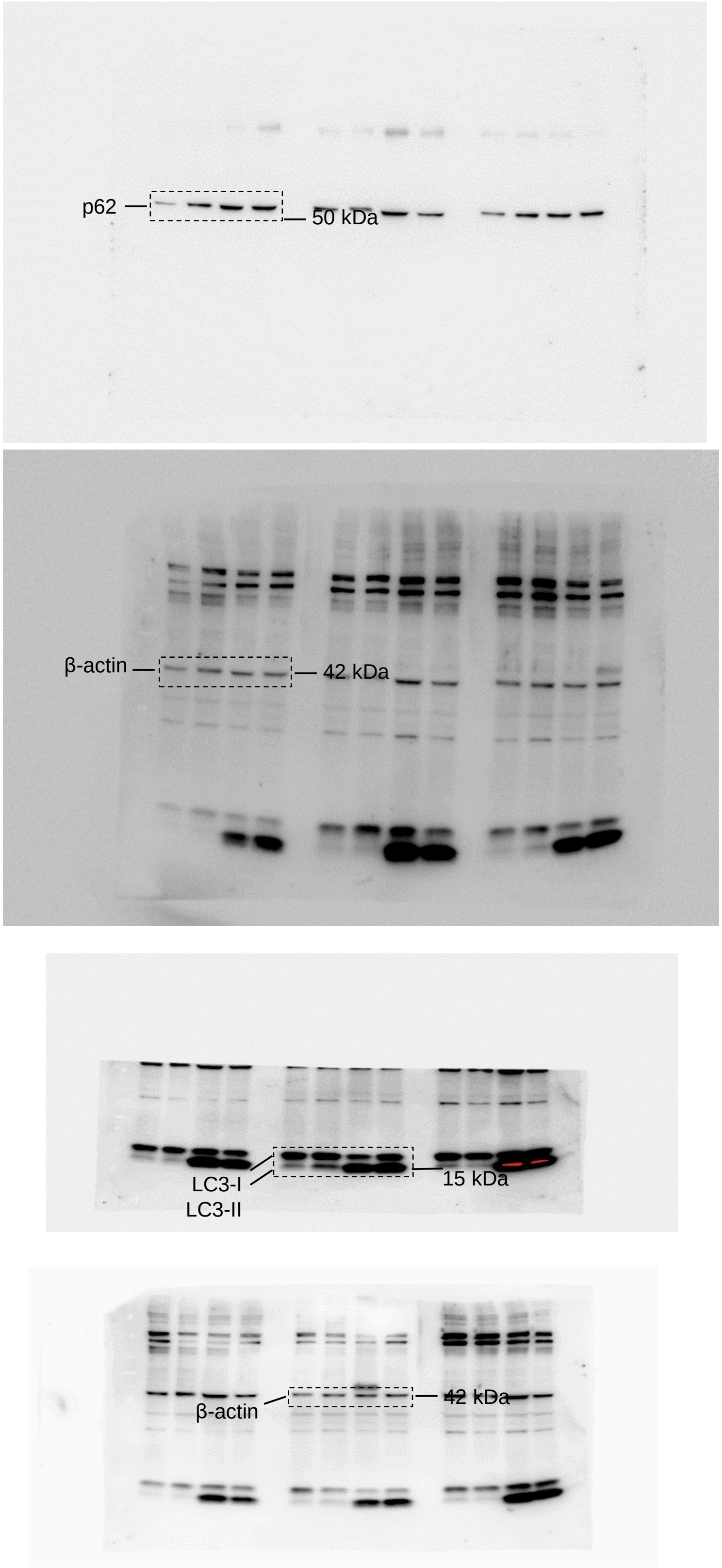

Supplementary Figure 19

a

Figure 5G, from left to right, first panels (cerebral cortex)

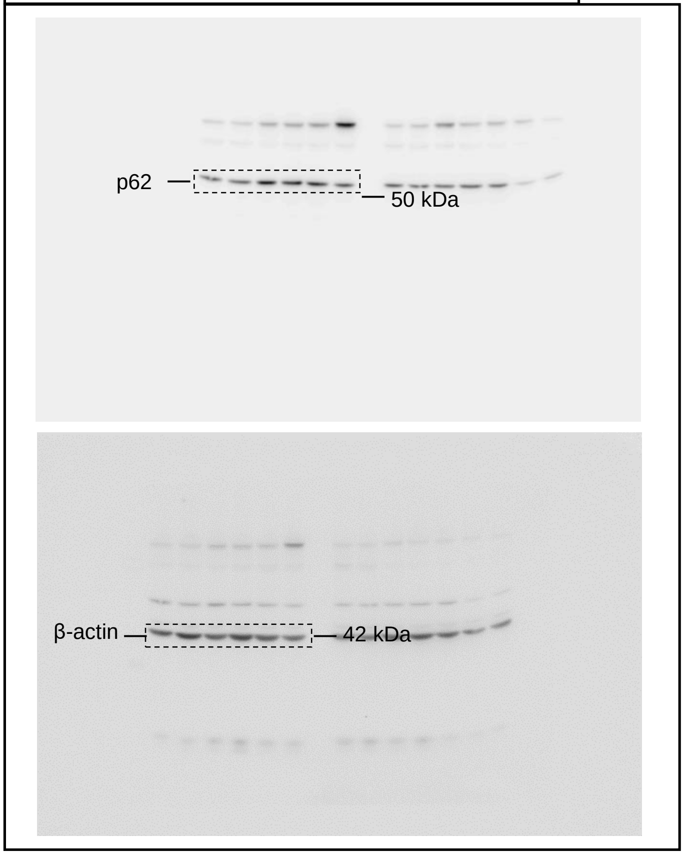

b

Figure 5G, from left to right, second panels (hippocampus)

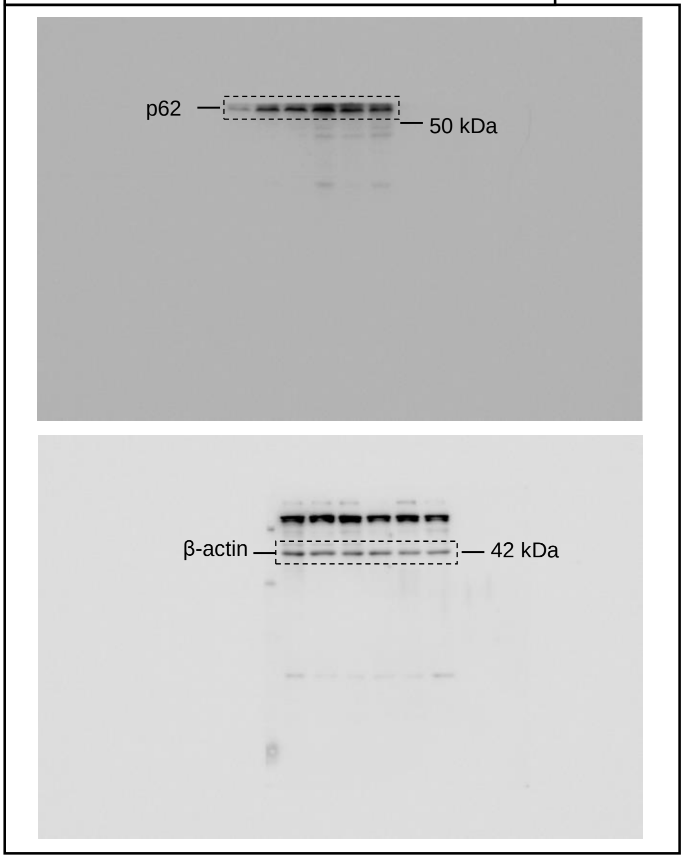

Supplementary Figure 20

a

Figure 5G, from left to right, third panels (cerebral cortex)

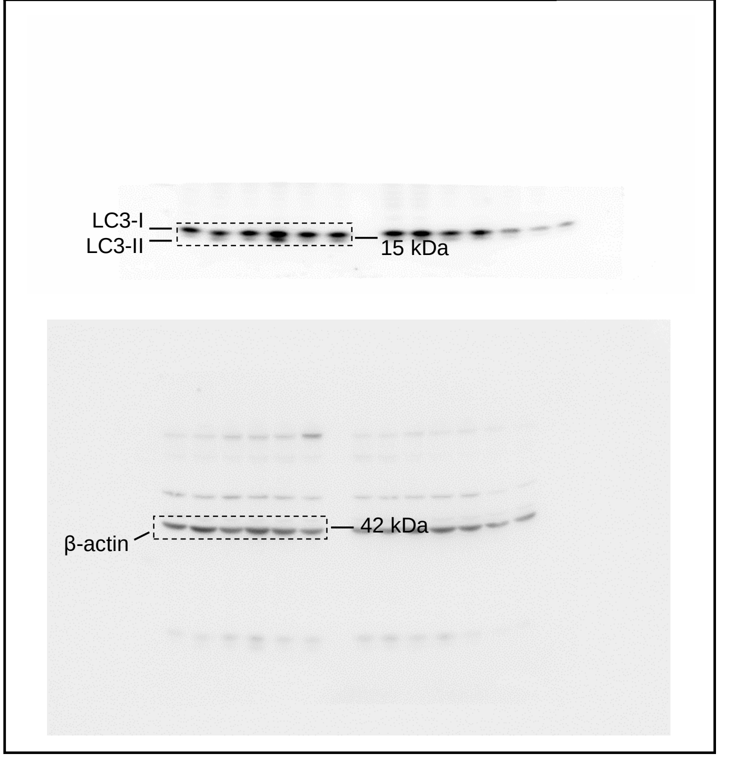

b

Figure 5G, from left to right, fourth panels (hippocampus)

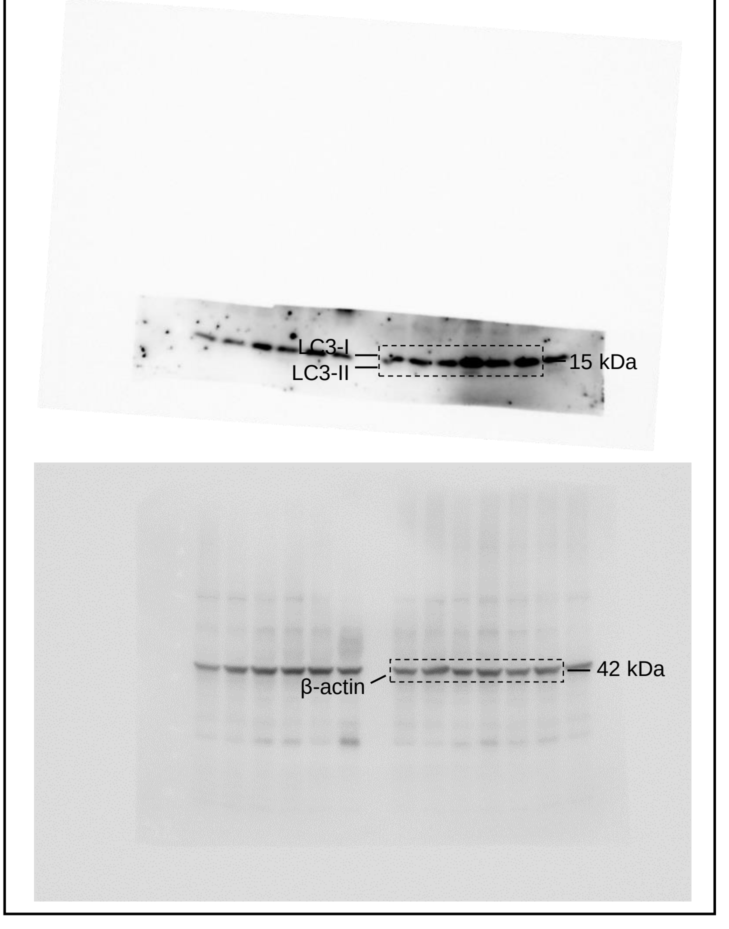

Supplementary Figure 21

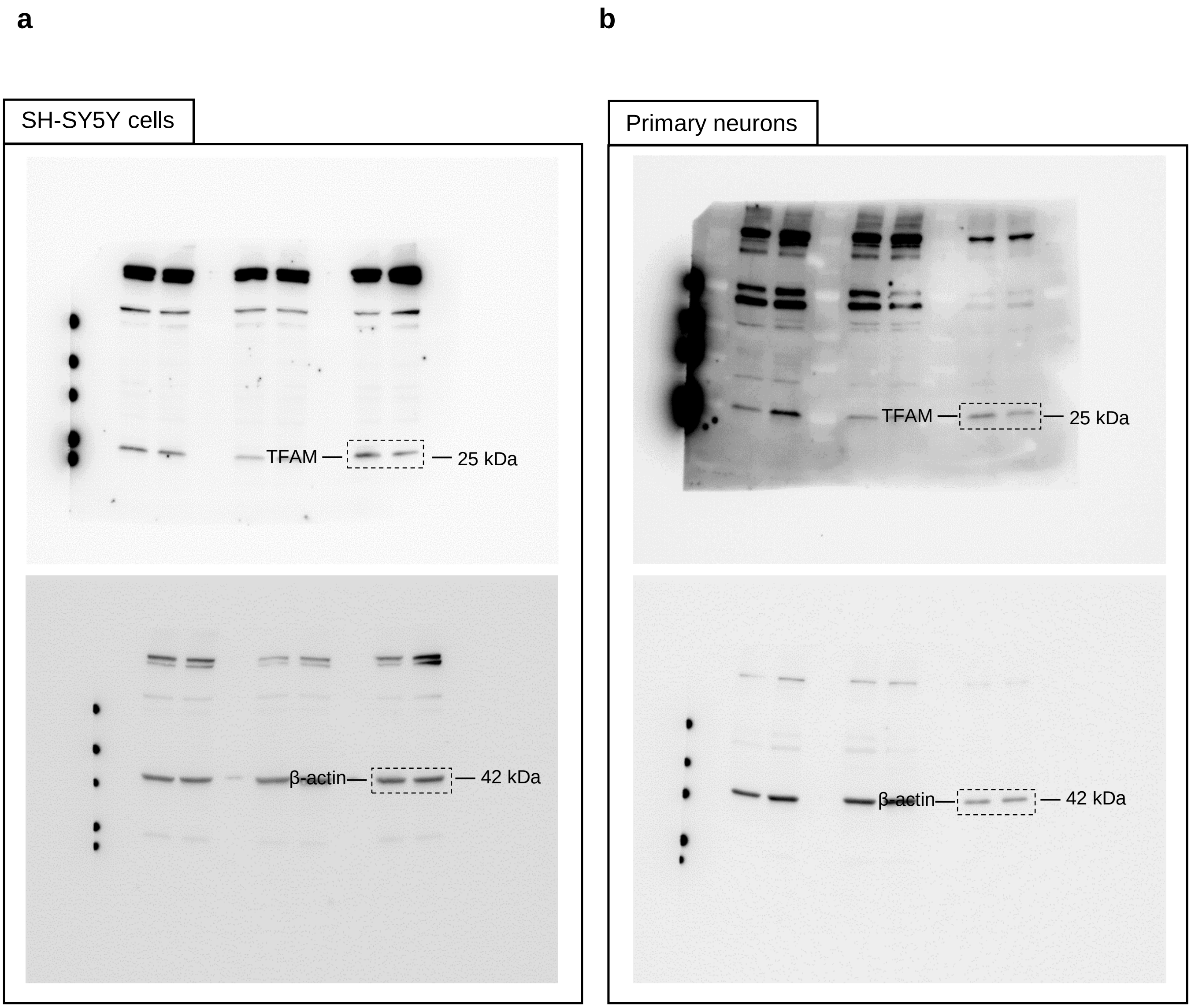

Supplementary Figure 22
